# Binding of the Fkh1 Forkhead Associated Domain to a Phosphopeptide within the Mph1 DNA Helicase Regulates Mating-Type Switching in Budding Yeast

**DOI:** 10.1371/journal.pgen.1006094

**Published:** 2016-06-03

**Authors:** Antoinette M. Dummer, Zhangli Su, Rachel Cherney, Koyi Choi, John Denu, Xiaolan Zhao, Catherine A. Fox

**Affiliations:** 1 Department of Biomolecular Chemistry, School of Medicine and Public Health, University of Wisconsin-Madison, Madison, Wisconsin, United States of America; 2 Graduate Program in Cellular and Molecular Biology, University of Wisconsin-Madison, Madison, Wisconsin, United States of America; 3 Wisconsin Institute for Discovery, University of Wisconsin-Madison, Madison, Wisconsin, United States of America; 4 Molecular Biology Program, Memorial Sloan Kettering Cancer Center, New York, New York, United States of America; Cornell University, UNITED STATES

## Abstract

The *Saccharomyces cerevisiae* Fkh1 protein has roles in cell-cycle regulated transcription as well as a transcription-independent role in recombination donor preference during mating-type switching. The conserved FHA domain of Fkh1 regulates donor preference by juxtaposing two distant regions on chromosome III to promote their recombination. A model posits that this Fkh1-mediated long-range chromosomal juxtaposition requires an interaction between the FHA domain and a partner protein(s), but to date no relevant partner has been described. In this study, we used structural modeling, 2-hybrid assays, and mutational analyses to show that the predicted phosphothreonine-binding FHA domain of Fkh1 interacted with multiple partner proteins. The Fkh1 FHA domain was important for its role in cell-cycle regulation, but no single interaction partner could account for this role. In contrast, Fkh1’s interaction with the Mph1 DNA repair helicase regulated donor preference during mating-type switching. Using 2-hybrid assays, co-immunoprecipitation, and fluorescence anisotropy, we mapped a discrete peptide within the regulatory Mph1 C-terminus required for this interaction and identified two threonines that were particularly important. *In vitro* binding experiments indicated that at least one of these threonines had to be phosphorylated for efficient Fkh1 binding. Substitution of these two threonines with alanines (*mph1-2TA*) specifically abolished the Fkh1-Mph1 interaction *in vivo* and altered donor preference during mating-type switching to the same degree as *mph1*Δ. Notably, the *mph1-2TA* allele maintained other functions of Mph1 in genome stability. Deletion of a second Fkh1-interacting protein encoded by *YMR144W* also resulted in a change in Fkh1-FHA-dependent donor preference. We have named this gene *FDO1* for **F**orkhead one interacting protein involved in **do**nor preference. We conclude that a phosphothreonine-mediated protein-protein interface between Fkh1-FHA and Mph1 contributes to a specific long-range chromosomal interaction required for mating-type switching, but that Fkh1-FHA must also interact with several other proteins to achieve full functionality in this process.

## Author Summary

Specific chromosomal interactions between distal regions of the genome allow for DNA transactions necessary for normal cell function, but the protein-protein interfaces that regulate such interactions remain largely unknown. The budding yeast Fkh1 protein uses its evolutionarily conserved phosphothreonine-binding FHA domain to regulate a long-range DNA transaction called mating-type switching that allows yeast cells to switch their sexual phenotype. In this study, another conserved nuclear protein, the Mph1 DNA repair helicase, was shown to interact directly with the FHA domain of Fkh1 to regulate mating-type switching. The Fkh1-Mph1 interaction required two phosphorylated threonines on Mph1 that were dispensable for many other Mph1-protein interactions and other Mph1 chromosomal functions. Thus a discrete protein-protein interface between two multifunctional chromosomal proteins helps define a long-range chromosomal interaction important for controlling cell behavior.

## Introduction

The *Saccharomyces cerevisiae* Fkh1 (forkhead homolog 1) protein is a member of the FOX (forkhead box) family of proteins defined by their winged-helix DNA binding domains. The FOX family proteins are best known for their transcriptional roles in regulating the cell cycle and differentiation [1]. For example, the Fkh1 paralog, Fkh2, controls the cell-cycle regulated transcription of the *CLB2*-cluster genes required for the proper execution of M-phase events [2–12]. Fkh1 appears to play an accessory role here, as deletion of both *FKH1* and *FKH2*, but not either gene alone, causes severe cell-cycle dysfunction. However, its molecular functions and the mechanisms by which Fkh1 participates in this process remain poorly understood [3,13]. Accumulating evidence indicates that Fkh1 and 2 also play a transcription-independent role in regulating the timing profile for DNA replication origin activation [14,15]. In addition, Fkh1 has a unique role not shared with Fkh2 in recombination-mediated mating-type switching [16,17], but the molecular mechanisms of this Fkh1 function are not completely understood.

**Fig 1:**
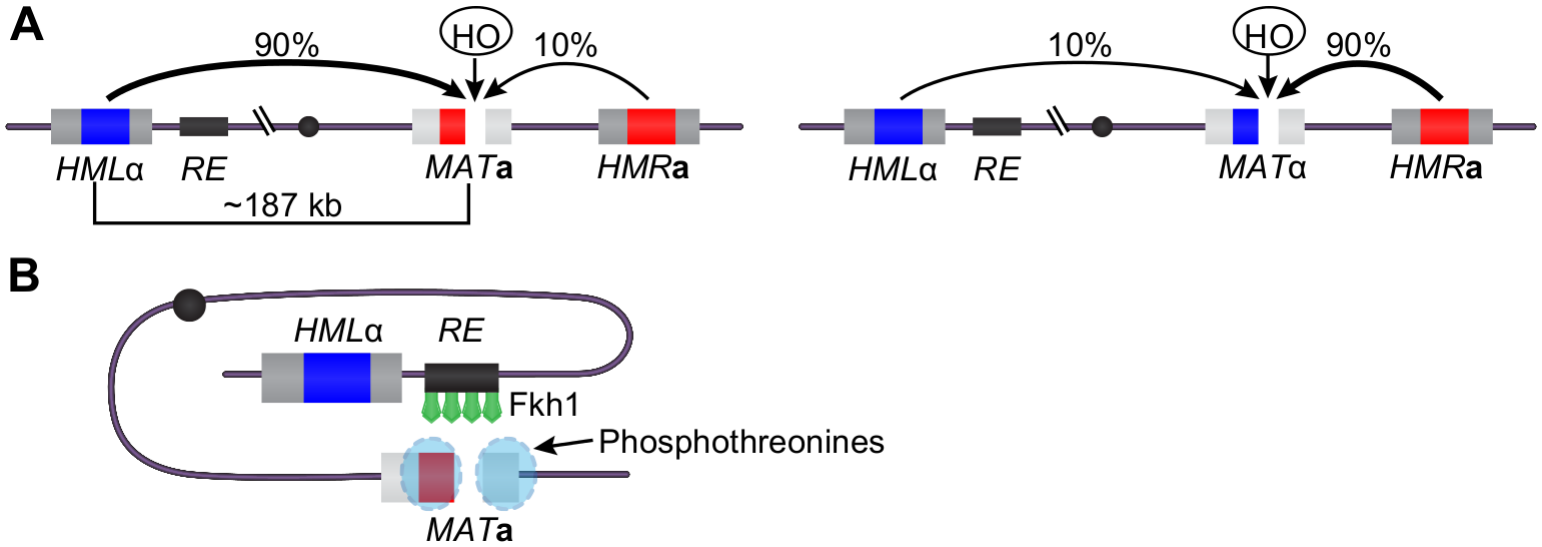
Donor preference during mating-type switching is regulated by Fkh1 through the recombination enhancer. (**A**) *MAT***a** cells primarily use *HML*α as a donor, while *MAT*α cells use *HMR***a** as a donor, resulting in a high frequency of mating-type switching. Adapted from [17,23]. (**B**) Model of Fkh1-regulated mating-type switching. Adapted from [22]. The HO endonuclease cuts at *MAT***a**, and a protein(s), phosphorylated on specific threonine residues binds to the double-strand break (DSB). Fkh1 bound to the RE interacts with these phosphorylated proteins. These interaction(s) bring *HML* close to the DSB at *MAT* and result in substantial preference for recombination between *MAT* and *HML*.

Mating-type switching allows haploid cells of one mating-type to switch to the other, consequently enabling two neighboring haploids to mate and undergo sexual reproduction [18]. Mating-type switching is a critical aspect of yeast biology and evolution that has been used as a model to better understand the repair of double-strand breaks (DSBs) through homologous recombination [19]. During mating-type switching, a DSB is generated by the HO endonuclease at the *MAT* locus that contains either **a**- or alpha- mating-type genes. This break is repaired through homologous recombination using donor template sequences located at the silent mating-type loci, *HML* or *HMR*, at the opposite ends of the same chromosome as *MAT* (Fig 1A) [19,20]. *HML* and *HMR* contain a repressed copy of alpha (*HML*α) or **a** genes (*HMR***a**), respectively. Productive mating-type switching requires the proper choice between these two donor loci so that the opposite mating-type gene is inserted at *MAT*. Thus *MAT***a** cells favor recombination with *HML*α ~90% of the time, while *MAT*α cells choose *HMR***a** as a donor ~90% of the time (Fig 1A). The choice of mating-type donor, that is the directionality of mating-type switching, does not depend on the mating-type genes themselves, but on the protein-DNA complex that forms at a regulatory *cis*-element called the recombination enhancer (RE), a chromosomal region located between the *MAT* and *HML* loci [21]. Fkh1 has been shown to regulate the directionality of mating-type switching by binding to RE in *MAT***a** cells and establishing a strong preference for *HML*α for repair (Fig 1B) [16]. The forkhead associated (FHA) domain of Fkh1 is sufficient for this function as a LexA-Fkh1-FHA domain fusion is fully functional in regulating donor preference if RE is replaced with LexA binding sites [22].

FHA domains are present in many proteins involved in chromosomal functions and serve as protein-protein interaction modules that specifically recognize phosphorylated threonine residues [24–28]. This property of FHA domains and the involvement of the Fkh1 FHA domain in donor preference during mating-type switching support a model in which the Fkh1 FHA domain controls the directionality of mating-type switching through direct interactions with a phosphorylated protein partner(s) (Fig 1B). This model posits that the presumed partner protein(s) likely binds the DSB at *MAT***a**, and through an interaction with Fkh1 bound to RE, localizes *HML*α, the donor locus, near the DSB, allowing for efficient strand invasion to occur [22]. Currently, the identities of this Fkh1 partner protein(s) is unknown, and the possible roles of this protein(s), or the Fkh1 FHA domain, in Fkh1’s other cellular roles are also unknown.

To address these issues, we performed a 2-hybrid interaction screen that identified five Fkh1-interacting proteins. Domain analyses revealed that Fkh1 interacted with each of these proteins via its FHA domain. Mutation of key residues within this domain revealed that it was important for Fkh1’s role in cell-cycle regulation, though no single interacting partner could account for this role. In addition, our genetic analyses indicate that functions of the FHA domain outside of its phosphopeptide binding activity contribute to Fkh1’s cell cycle role. Focusing on one Fkh1 binding partner, Mph1, we found that its loss altered donor preference during mating-type switching. Using multiple approaches, we defined a peptide within Mph1 that interacted directly and efficiently with purified Fkh1 *in vitro* and in a manner that depended on the phosphorylation state of two threonines within the peptide. Mph1 also interacted with Fkh1 in cells and this interaction required the same threonines that mediated the Fkh1-Mph1-peptide interaction. Alanine substitutions of the two threonines in Mph1 (*mph1-2TA*) caused a defect in donor preference during mating-type switching similar to that caused by *mph1*Δ. However, *mph1-2TA* cells did not share other cellular defects caused by *mph1*Δ, such as sensitivity to MMS or an elevated rate of mutation. Because *MPH1* could only partially explain Fkh1-FHA’s role in mating-type switching, we examined the role of a second Fkh1-interacting protein identified in our screen, encoded by *YMR144W*. A *ymr144W*Δ also altered mating-type switching directionality, and *ymr144W*Δ *mph1*Δ reduced the efficiency of this process beyond that of either mutation alone. We have named this gene *FDO1* for **F**orkhead one interacting protein involved in **do**nor preference. Thus we have delineated a specific cellular role for Fkh1 and Mph1 mediated by an FHA-phosphothreonine interaction, and provided evidence that Fkh1-FHA bound to the RE likely must recognize several proteins at the DSB for full function in mating-type switching directionality.

## Results

### Yeast 2-hybrid screen identified five proteins that interact with Fkh1

To identify proteins that interact with Fkh1, we used a 2-hybrid interaction screen in which a Fkh1-Gal4 DNA binding domain (Fkh1-GBD) fusion protein served as bait and a library of Gal4 activation domain (GAD) fusions served as prey [29]. This Fkh1-GBD fusion protein contained the entire Fkh1 coding sequence except for its forkhead DNA binding domain, as this domain was replaced with GBD. Five proteins were identified as positive interactors from this screen (Table 1). These included the DNA helicase Mph1 that is involved in recombinational repair, the Gln3 and Ure2 proteins involved in transcriptional control, and the two uncharacterized proteins with unclear functions [30–34]. Mph1, Ure2, and Fdo1 (formerly Ymr144w) were identified in a previous proteomic screen as proteins that co-purified with a Fkh1-FLAG fusion protein [35], verifying the effectiveness of our screen.

**Table 1:** Fkh1 2-hybrid interacting proteins.

Name	Description	Region identified in screen (aas)	Fkh1-FLAG interactor? [35]
*MPH1*	DNA repair helicase	762–993	Yes
*ECM30*	Putative protein involved in cell wall biosynthesis	1005–1183	No
*GLN3*	Transcriptional activator of genes regulated by nitrogen catabolite repression	20–189	No
*URE2*	Transcriptional regulator that acts by inhibition of GLN3 transcription in good nitrogen source	84–354	Yes
*FDO1 (YMR144W)*	Putative nuclear protein of unknown function	98–342	Yes

### The FHA domain of Fkh1 interacted with the C-terminal domain of Mph1

**Fig 2:**
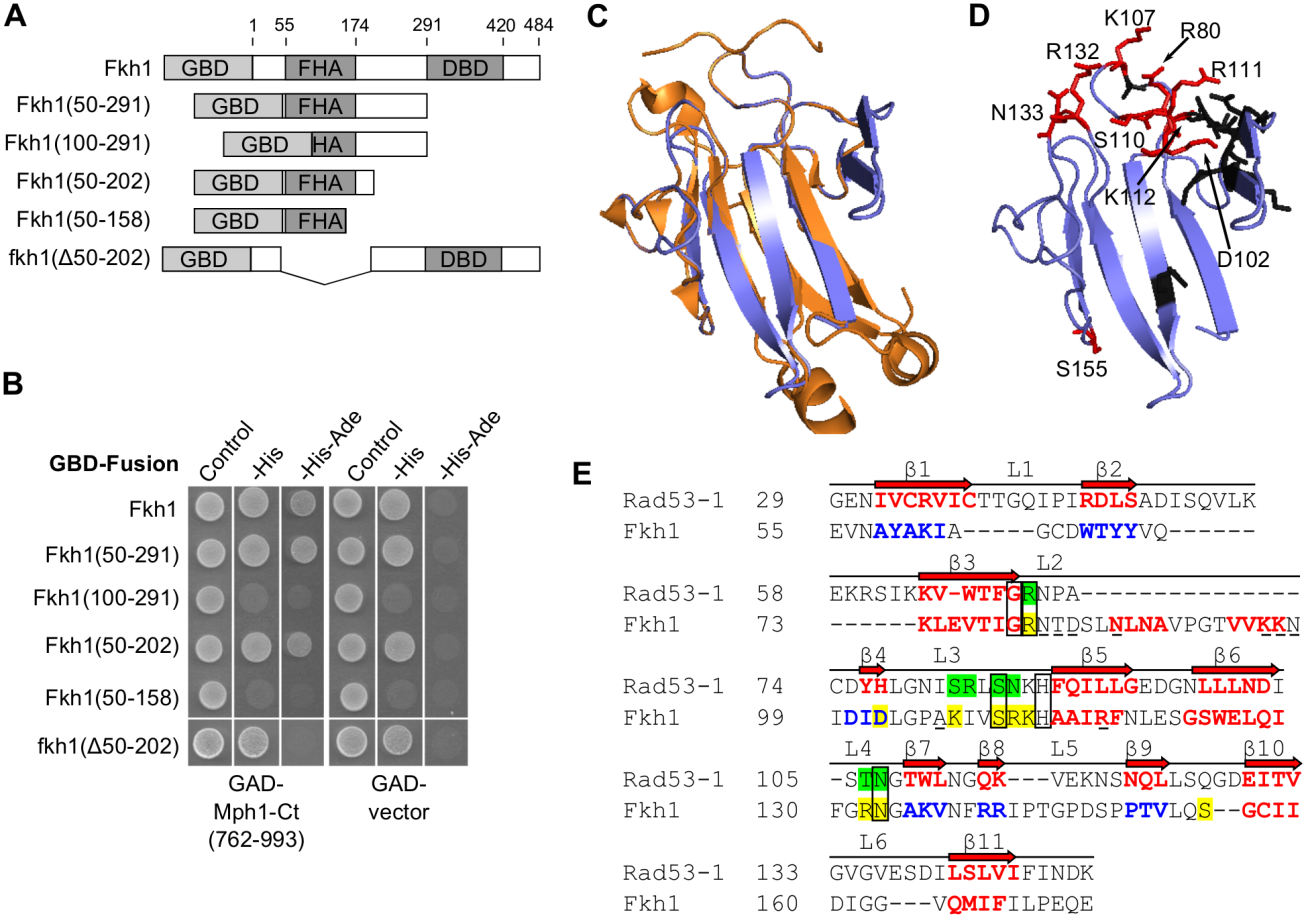
Fkh1 interacted with Mph1 through the FHA domain. (**A**) Diagram of GBD-Fkh1 fusions used in panel (B). (**B**) Yeast 2-hybrid assays performed using various regions of Fkh1 as bait (shown in panel (A)). Interaction was assessed by selection for activation of reporter genes *HIS3* and *ADE2* on media lacking histidine or both adenine and histidine. Note that many GBD-Fkh1 fusion proteins were able to activate transcription of the *HIS3* reporter gene in the absence of an interaction partner. Therefore, interaction was defined as the ability to grow on selective media only in the presence of an interaction partner, and not the GAD alone. For most constructs analyzed, interaction was determined by growth on media lacking both histidine and adenine. (**C**) A predicted structure for amino acids 72–170 of the Fkh1 FHA domain was generated by SWISS-MODEL using the N-terminal Rad53 FHA domain as a template (PDB 1G6G) [42–45]. See S1 Fig for a table of other templates used for homology modeling. The Rad53 structure with associated peptide is shown in orange and was chosen for this comparison because it produced the highest quality model (as determined by QMEAN Z-scores-see S1 Fig). The predicted Fkh1 FHA domain structure is shown in blue. (**D**) Predicted structure for the Fkh1 FHA domain. Residues analyzed that were required for binding Mph1 in the 2-hybrid context are shown in red and labeled by their residue number. Residues analyzed that were not required for binding Mph1 are shown in black (See S2 Fig for 2-hybrid data). (**E**) Structure-based sequence alignment between the N-terminal Rad53 FHA domain (Rad53-1) and the Fkh1 FHA domain (Fkh1). Alignment was generated using structural predictions of the Fkh1 FHA domain, which include a combination of the Rad53-based homology model and secondary structure prediction [46]. Conserved FHA domain β-strands and the loops which comprise the peptide binding site are labeled [47]. Rad53 β-strands are shown in red. Fkh1 β-strands predicted in the homology model are shown in red and those predicted from secondary structure prediction are shown in blue. The five conserved FHA residues that comprise the phosphothreonine binding pocket are boxed. Fkh1 residues shown to be required for binding to Mph1 are highlighted yellow and residues from Rad53 that make direct contact with a Rad9-derived peptide are highlighted green. Fkh1 residues that were tested but not required for Mph1 binding are underlined.

To define how Fkh1 interacts with the proteins identified in our screen, we tested which regions of Fkh1 interacted with Mph1, the yeast homolog of the human FANCM helicase [36,37]. The Fkh1-Mph1 interaction was of particular interest because both proteins are implicated in recombinational repair, though each protein also has other functions [16,19,22,36,37]. Our 2-hybrid screen identified the C-terminal region of Mph1 (amino acids 762–993, henceforth referred to as Mph1-Ct), which has been shown to act as a regulatory domain on this protein, providing interaction sites for numerous proteins that regulate its function [38–41]. To define the region of Fkh1 that interacts with Mph1-Ct, we tested several GBD constructs containing different regions of Fkh1 (Fig 2A) and found that amino acids 50–202 of Fkh1, the majority of which is comprised of the FHA domain, was sufficient for interaction with Mph1-Ct (Fig 2B). Conversely, the fkh1(Δ50–202) mutant did not interact with Mph1-Ct. Thus, the region of Fkh1 containing amino acids 50–202 (henceforth referred to as Fkh1-FHA) was necessary and sufficient to interact with Mph1-Ct.

### Modeling of the FHA domain of Fkh1 defined residues predicted to be important for phosphothreonine binding

Next, we examined whether the predicted phosphothreonine binding ability of Fkh1-FHA was required for binding Mph1. To this end, we performed homology modeling (Fig 2C and 2D) using published structures of multiple FHA domains as template (see S1 Fig). Of the homology models generated, the one using the well-characterized N-terminal FHA domain of the checkpoint protein Rad53 [42] as template yielded the highest quality model (S1 Fig). Using this information, as well as additional secondary structure prediction [46] of the regions not modeled, we generated a structure-based sequence alignment of the Fkh1 and Rad53 FHA domains. Upon generation of the homology model and alignment, we found that the FHA domain of Fkh1 is ~50 amino acids larger than previous studies have reported [5,22], as it contains two extra predicted β-strands in addition to the 11 β-strands which comprise the core FHA domain fold [47] (Fig 2C–2E). In addition, this approach allowed for identification of several amino acids predicted to be on or near the phosphopeptide binding surface of Fkh1 (Fig 2D, homology model, and Fig 2E, structure-guided alignment). Five of these residues (Fig 2E, boxed) form the phosphothreonine binding pocket and are conserved among FHA domains [48]. In addition, multiple residues within loops two, three, and four of this domain can make direct contacts with phosphopeptide binding partners in other FHA domains and are less well conserved, allowing different FHA domains to have distinct binding specificities [47]. We note that the predicted phosphopeptide binding surface of Fkh1 FHA is predominantly positively charged, suggesting a preference for binding to a peptide with negatively charged residues (S3 Fig).

### Putative phospho-binding residues of the Fkh1 FHA domain were important for associating with Mph1

Based on this structural and alignment information we engineered several single amino acid substitutions in Fkh1-FHA and assessed their ability to interact with Mph1-Ct in 2-hybrid assays. We found that several amino acids predicted to be on the phosphopeptide binding surface, as well as a more distal residue (S155), were important for interaction with Mph1 (Fig 2D-red, Fig 2E-highlighted yellow and S2A Fig). For example, Fkh1 R80 is conserved in all FHA domains and the analogous residue in Rad53 makes direct contact with its partner peptide [42,48]. Substitution of alanine for Fkh1 R80 abolished the interaction between Fkh1-FHA and Mph1-Ct (Fig 2D and 2E and S2A Fig). In contrast, amino acid substitutions in several amino acids predicted not to be on the phosphopeptide binding interface of Fkh1-FHA had no effect on the Fkh1-FHA-Mph1-Ct 2-hybrid interaction, including substitutions within the extended loop two (Fig 2D-black, Fig 2E-underlined, and S2B Fig). Taken together, these mutagenesis studies suggest that the predicted phosphopeptide-interaction surface of the FHA domain of Fkh1 is important for interaction with Mph1.

### Fkh1 interacted with five partner proteins via its conserved FHA domain

To test whether the FHA domain of Fkh1 is also involved in interacting with other proteins recovered from our 2-hybrid screen, we examined their binding to Fkh1-FHA and the mutant constructs described above in the 2-hybrid assay (S2A and S2C Fig). Fkh1-FHA was necessary and sufficient to interact with Ecm30(1005–1183), Gln3(20–189) and Ure2(84–354) (S2A Fig). In addition, with only a few exceptions for assays with Gln3, the amino acid substitutions that abolished Fkh1-FHA-Mph1-Ct binding also abolished the interaction with these other proteins. Finally, a region containing the FHA domain of Fkh1 was necessary but not sufficient to interact with Fdo1, suggesting the involvement of additional regions for their interaction (S2C Fig). Thus Fkh1 can interact with a number of distinct proteins via its conserved FHA domain.

### The FHA domain contributed to Fkh1’s overlapping role with Fkh2 in the regulation of cell growth

**Fig 3:**
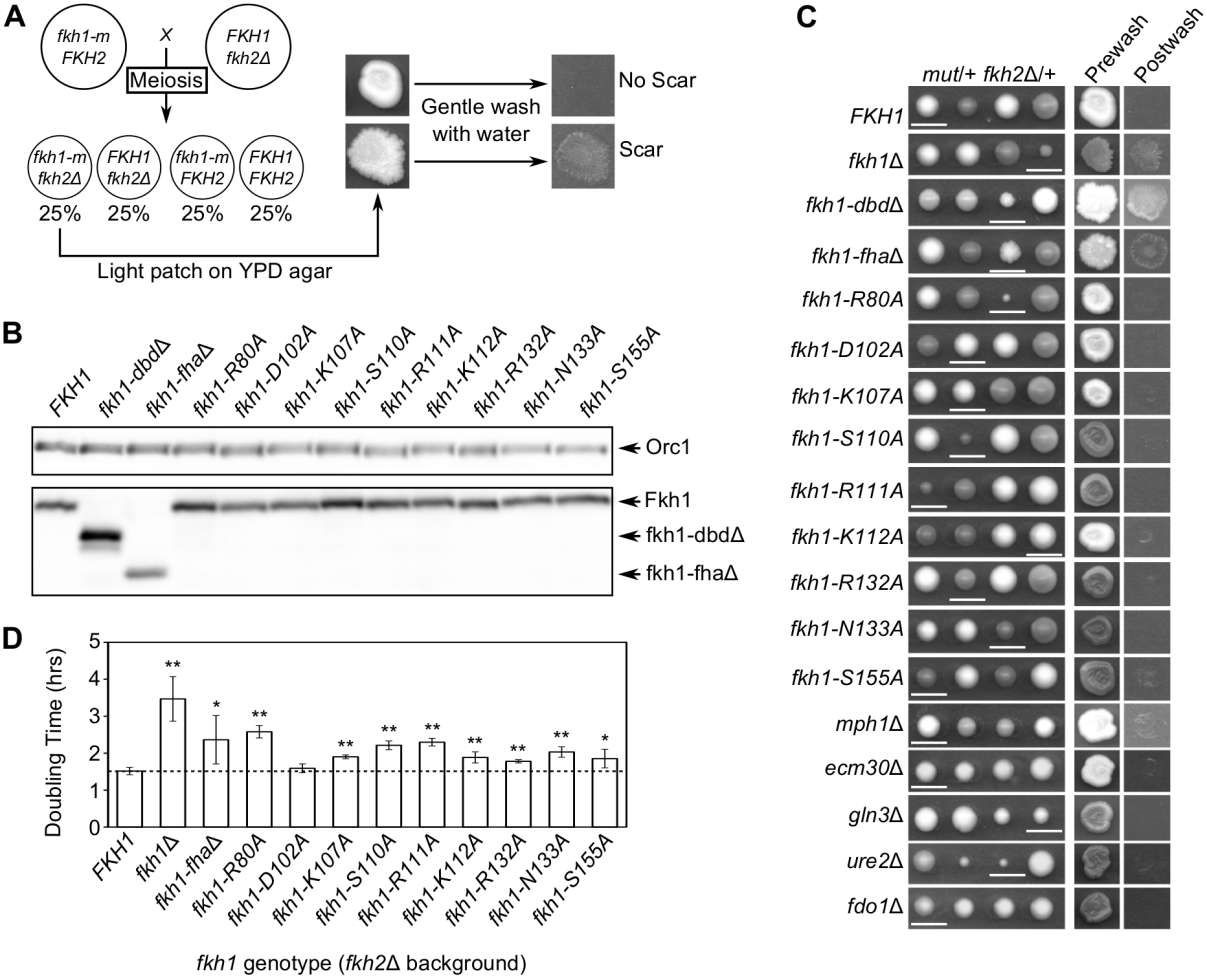
The FHA domain of Fkh1 was involved in maintaining proper cell morphology and growth rate. (**A**) Schematic of morphology experiment. Haploids expressing the indicated mutant were crossed to *fkh2*Δ haploid yeast to form heterozygous *fkh1-m*/+ *fkh2*Δ/+ diploids. Diploids underwent meiosis and tetrads were dissected. Spore clones containing both the indicated mutation and *fkh2*Δ were assessed for morphology differences by agar scarring assays. All pertinent genotypes were assessed by PCR. Agar scarring was assessed by gently patching haploid strains onto YPD and washing with H_2_O after three days. (**B**) Protein immunoblot of *fkh1-m* strains using anti-Fkh1 antibody. Orc1 detected with an anti-Orc1 antibody [49] served as a loading control. (**C**) Morphology assays as described in panel (A). Representative tetrads in which all four possible allelic combinations are present are shown. Spore clones containing both the indicated mutation and *fkh2*Δ are underlined. Agar scarring assays were performed on the underlined spore. (**D**) Doubling times of *fkh1-m fkh2*Δ strains in liquid YPD media. Averages are based on at least 3 replicates. Error bars represent 1 standard deviation. Asterisks indicate level of statistical significance compared to *fkh2*Δ cells. P-value of 0.05–0.001 = *, p-value <0.001 = **.

To understand the biological functions of protein interactions observed with the Fkh1 FHA domain, we investigated whether this domain was required for the functions shared between Fkh1 and 2, namely the regulation of the cell cycle and colony morphology. Deletion of both *FKH1* and *FKH2*, but not either gene alone, causes cell-cycle dysfunction that leads to a pseudohyphal-like growth that produces rough, chalky colonies that scar solid agar medium [3–7]. While the FHA domain of Fkh2 is important for *FKH2* function [9,10], the role of the Fkh1 FHA domain in *FKH1* function in these phenotypes has not been reported. Therefore, we determined whether mutant versions of Fkh1 examined above (referred to as *fkh1-m*) resulted in these defects in a *fkh2*Δ background (Fig 3A). We note that all the examined *fkh1-m* proteins were expressed at levels similar to that of wild type Fkh1 (Fig 3B), indicating that any observed defects are not due to a loss of Fkh1 protein.

By examining spore clones generated from diploids heterozygous for both *fkh1-m* and *fkh2*Δ, we first confirmed previous findings that *fkh1*Δ *fkh2*Δ and *fkh1-dbd*Δ *fkh2*Δ yeast grew slowly and produced a colony that scarred the agar medium (Fig 3C) [3]. We also found that a *fkh1* allele lacking the FHA domain coding region (Δ50–202, *fkh1-fha*Δ), when combined with *fkh2*Δ, produced the same phenotype as *fkh1*Δ and *fkh1-dbdΔ* (Fig 3C). Thus, this N-terminal region including the Fkh1 FHA domain (residues 50–202) was important for Fkh1’s role in cell cycle regulation. The single residue substitution alleles examined, *fkh1-R80A*, *fkh1-S110A* and *fkh1-R111A* produced smaller colonies when combined with *fkh2*Δ, indicating that these single amino acids were also essential for wild-type Fkh1 function in this assay (Fig 3C). Each of these residues is predicted to be critical for the phosphopeptide binding function of the Fkh1 FHA domain. The remainder of the *fkh1-m* alleles examined in this assay caused no discernible defect when combined with *fkh2*Δ (Fig 3C). However, most of the alleles did reduce mitotic growth rates in liquid culture when combined with *fkh2*Δ, suggesting a defect in functions that overlap with Fkh2 (Fig 3D). The different effects of *fkh1*-fhaΔ versus the *fkh1-m* alleles suggest that Fkh1 residues 50–202 have functions beyond phosphopeptide binding activity in cell cycle regulation. Regardless, most single amino acid substitutions predicted to reduce or abolish FHA phosphopeptide binding activity caused mitotic growth defects, supporting a role for the Fkh1 FHA domain in Fkh1’s overlapping roles with Fkh2 in the yeast cell cycle.

### Fkh1’s overlapping role with Fkh2 did not depend on any single binding partner identified in the 2-hybrid screen

The data presented above supported the hypothesis that Fkh1’s role in cell-cycle regulation is mediated through the Fkh1 FHA domain’s interaction with one or more partner proteins. To test if any of the putative partners defined in the 2-hybrid screen were important for this role, we examined whether deletions of genes encoding these proteins phenocopied a *fkh1-fha*Δ or the *fkh1-m* alleles, such as *fkh1-R80A*, using the same genetic logic as in Fig 3A. A complete deletion of the protein coding regions for *MPH1*, *ECM30*, *GLN3*, *URE2* or *FDO1* did not reduce colony size when combined with a *fkh2*Δ, the diagnostic for Fkh1 function in this assay (Fig 3C). A *ure2*Δ did slow colony formation after dissection, but this effect did not require a *fkh2*Δ mutation. Therefore, no single Fkh1 interaction partner identified in the 2-hybrid screen could explain how the FHA domain contributed to Fkh1’s overlapping role with Fkh2 in cell-cycle regulation and morphology.

### The Fkh1-Mph1 interaction required either one of two specific threonines within the C-terminus of Mph1

**Fig 4:**
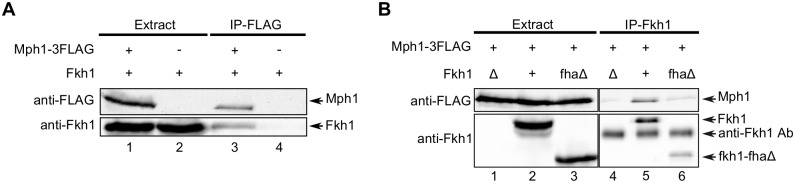
Fkh1 and Mph1 interact in yeast cell extracts. (**A**) Co-immunoprecipitation using anti-FLAG antibodies. Anti-FLAG antibodies were used to immunoprecipitate proteins from *MPH1-FLAG* (+) or *MPH1* (-) cells. The starting extract (extract) and immunoprecipitated proteins (IP) were then examined by protein immunoblotting using either anti-FLAG or anti-Fkh1 antibodies. (**B**) Anti-Fkh1 antibodies were used to immunoprecipitate proteins from *FKH1* (+), *fkh1*Δ (Δ), or *fkh1-fha*Δ (fhaΔ) cells and proteins were examined as described in panel (A).

**Fig 5:**
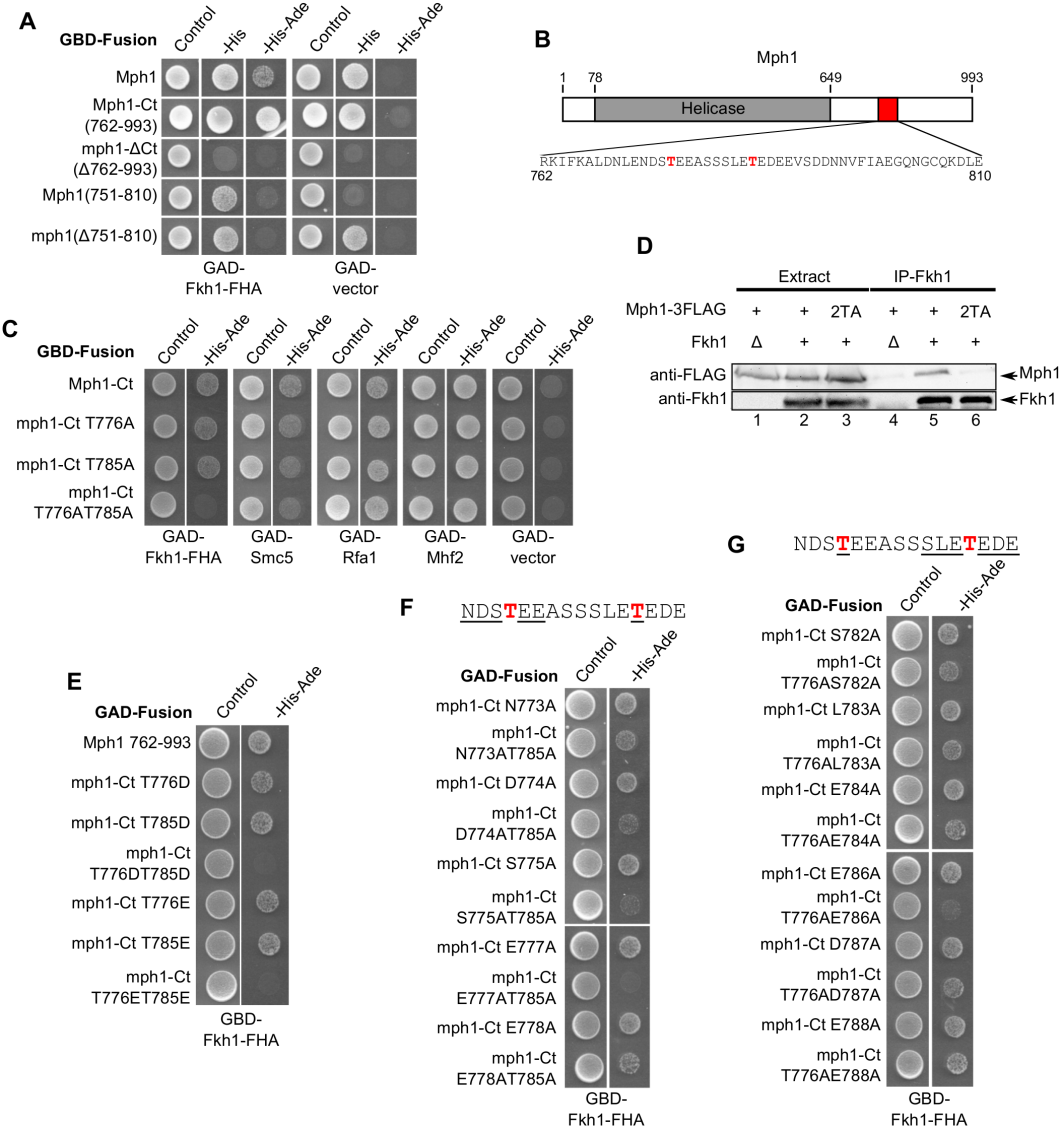
The Fkh1-Mph1 interaction required either one of two closely spaced threonines within the C-terminus of Mph1. (**A**) Yeast 2-hybrid assays performed using the indicated regions of Mph1 as bait. Note that many GBD-Mph1 fusion proteins were able to activate transcription of the *HIS3* reporter gene in the absence of an interaction partner. Therefore, interaction was defined as the ability to grow on selective media only in the presence of an interaction partner, and not the GAD alone. (**B**) Diagram of Mph1 primary structure [51]. The Mph1 region that interacts with Fkh1 occurs at the overlap between amino acids 751–810 and 762–993 (762–810, boxed in red) based on data in panel (A). The sequence of this region is displayed, with the two threonines it contains, T776 and T785, shown in red. (**C**) Yeast 2-hybrid assays using mutant forms of GBD-Mph1-Ct (amino acids 762–993) as bait and several known Mph1 interaction partners as prey. (**D**) Anti-Fkh1 antibodies were used to immunoprecipitate proteins from cells expressing, *MPH1-FLAG* (+) or *mph1-2TA-FLAG* (2TA) in *FKH1* (+) or *fkh1*Δ (Δ) backgrounds as described in Fig 4. (**E-G**) Yeast 2-hybrid assays using GBD-Fkh1-FHA (amino acids 50–202) and mutant forms of GAD-Mph1-Ct (amino acids 762–993) as indicated.

An important transcription-independent function of Fkh1 lies in the regulation of recombination-mediated mating-type switching [16,22]. Only one Fkh1-interaction partner identified in our 2-hybrid screen, Mph1, has an established role in recombinational repair [36,37,50]. Therefore, we focused on gaining a better molecular understanding of the Fkh1-Mph1 interaction. First, we confirmed this interaction using co-immunoprecipitation. Fkh1 was recovered in an immunoprecipitation with anti-FLAG antibodies only in cells expressing Mph1-FLAG (Fig 4A). Conversely, Mph1-FLAG was recovered in an immunoprecipitation with anti-Fkh1 antibodies only in cells expressing Fkh1 (Fig 4B). We found that this co-immunoprecipitation interaction depended on the region containing the FHA domain of Fkh1 (Fig 4B), validating our 2-hybrid results. In addition, 2-hybrid assays using different GBD-Mph1 fusions showed that amino acids 762–993 of Mph1 were both necessary and sufficient for its interaction with Fkh1-FHA, a result consistent with our finding in the original 2-hybrid screen (Fig 5A). Moreover, a smaller Mph1 fragment composed of amino acids 751–810 was sufficient to interact with Fkh1-FHA, albeit to a weaker extent than Mph1-Ct (amino acids 762–993), while Mph1 lacking this region was unable to bind the Fkh1 FHA domain (Fig 5A).

Previous studies of FHA domains [24,25,48] and the alignment and mutagenesis described in Fig 2 led to the prediction that the Fkh1 FHA domain binds partner proteins through contact with a phosphothreonine residue. To test this idea, we used the 2-hybrid assay to examine if any threonine in Mph1 was required for binding Fkh1. We focused on the overlapping 49 residues between Mph1(751–810) and Mph1(762–993), which contained only two threonines (Fig 5B). Substitution of alanine for both of these threonines (T776AT785A), but not either single T→A substitution, abolished the Mph1-Fkh1 interaction (Fig 5C). This finding was confirmed by co-immunoprecipitation, as Fkh1 failed to pull down mph1-T776AT785A in an immunoprecipitation experiment (Fig 5D). Both assays suggest that the Fkh1-Mph1 interaction required one of two threonines (T776 and T785) within Mph1. These residues are located within a highly acidic region of Mph1. The modeled structure of Fkh1-FHA showed a strongly positively charged concave surface, mainly formed by R80, K107, R111, K112, and R132 (S3 Fig), all of which were required for binding Mph1, suggesting Fkh1 uses this lysine-arginine-rich region to help recognize Mph1 through electrostatic interactions. The Mph1-Ct region serves as a regulatory hub on the Mph1 multifunctional helicase, directing its interactions with several partner proteins, including a subunit of the Smc5/6 complex (Smc5), the large subunit of RPA (Rfa1), and a subunit of the histone fold complex (Mhf2) [38–41]. To determine whether T776 and T785 were involved in these previously reported interactions, 2-hybrid assays were performed with the same series of Mph1 variants examined for interaction with Fkh1. Mph1-T776AT785A was able to interact with all three tested proteins (Fig 5C). Thus T776 and T785 directed a specific interaction between Fkh1 and Mph1 that was distinct from Mph1’s interaction with several other protein partners.

To better establish how Fkh1-FHA interacted with Mph1 we performed 2-hybrid assays in which T776 and/or T785 of Mph1 were replaced with aspartic acid or glutamic acid (Fig 5E). These negatively charged residues can act as phosphomimetics, and thus it was possible that if the role of these two threonine residues were fulfilled via their phosphorylation, that T→D or E substitutions would support the Fkh1-Mph1 2-hybrid interaction via electrostatic contributions alone. However, substitution of these threonines with aspartic acid or glutamic acid, but not the single substitutions, abolished interaction with Fkh1, indicating that T→D or E substitutions were as disruptive to the Fkh1-Mph1 interaction as the T→A substitutions we examined (Fig 5E). These data provide evidence that the threonine residue identities are particularly important, supporting the conclusion that the Fkh1 FHA domain is interacting with this region of Mph1 via classical FHA-phosphothreonine peptide contacts and not merely electrostatic interactions.

Many FHA domains (including the Rad53 N-terminal FHA domain) display a preference for particular amino acids at the pT +3 residue, while other FHA domains have a preference for particular amino acids at other positions [47,52]. As a first step toward understanding the binding preferences of the Fkh1 FHA domain we looked at how substitution of alanine for residues surrounding the two threonines in Mph1 affected Fkh1 binding. We found that substitution of alanine for any of these residues alone did not abolish Fkh1 binding, consistent with the finding that any single T→A substitution (T776A or T785A) did not abolish the Fkh1-Mph1 interaction. However, substitution of alanine for residues surrounding T776 in combination with a T785A substitution did reduce the Fkh1 2-hybrid interaction (Fig 5F). In particular, substitution of alanine for the aspartate at position 774, the serine at position 775, or the glutamate at position 777 in combination with T785A reduced or abolished the Fkh1-Mph1 interaction. Thus the region surrounding T776, including residues D774, S775 and E777, contributed to the Fkh1-Mph1 interaction. We used the same approach to define important residues surrounding T785, analyzing alanine substitutions in combination with T776A (Fig 5G). These data provided evidence that the region surrounding T785, most notably residue E786 but also to a lesser degree residue S782 and E784, contributed to the Fkh1-Mph1 interaction. These data provide additional evidence that this region of Mph1 contains two separate and independent FHA-binding motifs and that both motifs have similar features, including a preference for glutamic acid at the pT+1 position.

### Recombinant Fkh1 interacted directly with phosphorylated Mph1-derived peptides

**Fig 6:**
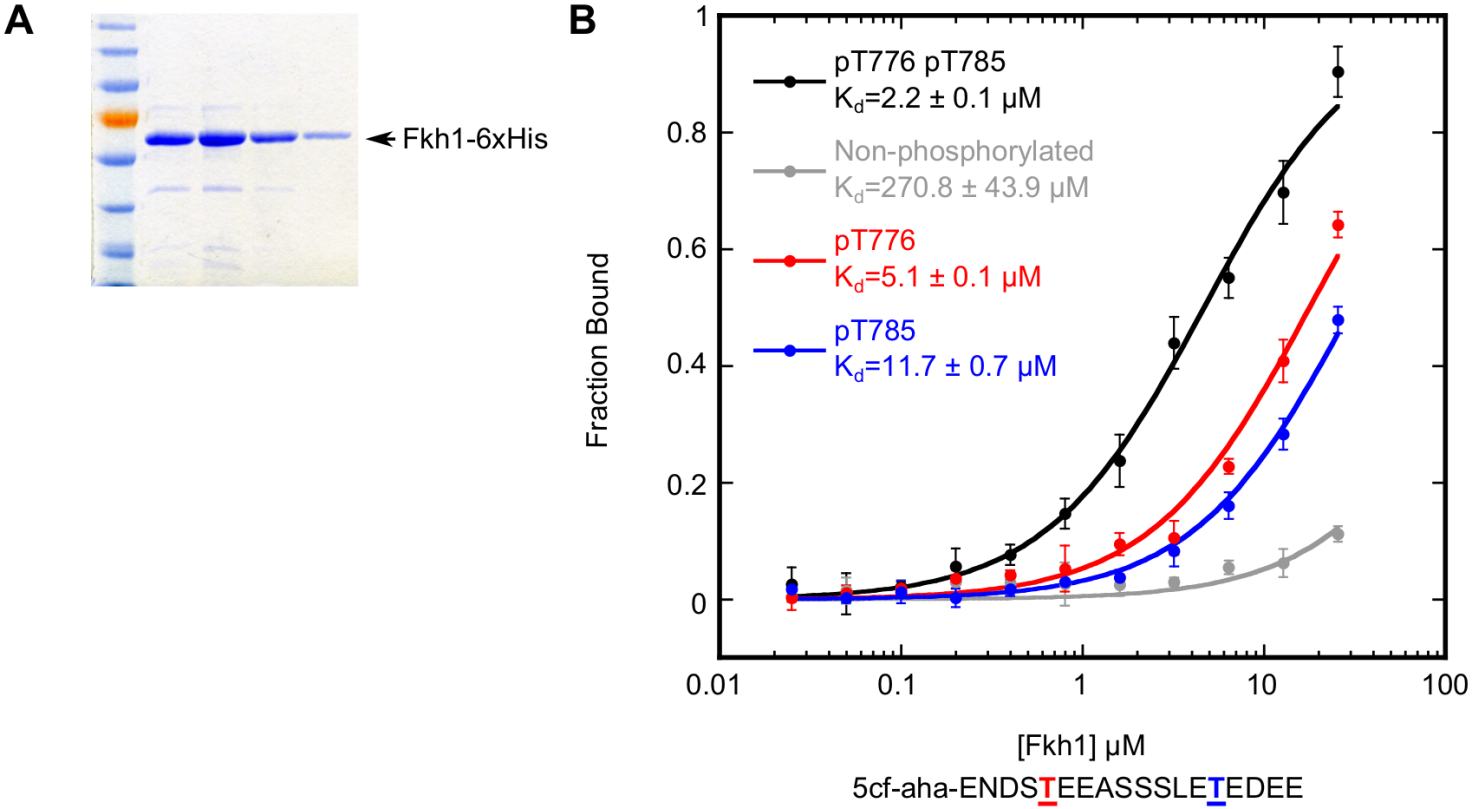
Recombinant Fkh1 directly bound to an Mph1-derived peptide in a phosphorylation-dependent manner. (**A**) Polyacrylamide gel showing recombinant Fkh1-6xHis protein used for peptide binding in (B). (**B**) Peptide binding assay of Fkh1-6xHis and Mph1-derived peptides using fluorescence anisotropy. Binding reactions contained the indicated concentration of purified His-tagged Fkh1 and 3 nM 5-carboxyfluorescein labeled Mph1-derived peptide (5cf-aha-ENDSTEEASSSLETEDEE) with threonines phosphorylated or unmodified, as indicated.

Next, we tested whether Fkh1 interacted directly with Mph1 through the region containing T776 and T785 and if this interaction was controlled by phosphorylation of these threonines. To this end, recombinant Fkh1-6xHis was purified from *E*. *coli* and its ability to bind an 18-residue peptide representing Mph1(772–789) was assessed by fluorescence anisotropy (Fig 6). The peptide that was phosphorylated on both T776 and T785 bound purified Fkh1 efficiently, with a K_d_ of 2.2 μM, well within range of other FHA-phosphopeptide interaction affinities [47]. The non-phosphorylated version of the peptide bound Fkh1 with a >100-fold reduced affinity (K_d_ of 270.8 μM). In addition, and consistent with the effects observed in the 2-hybrid assays in Fig 5C, mono-phosphorylated forms of the peptide (i.e. containing phosphorylation on only T776 or T785) also bound Fkh1, albeit with modestly reduced affinities. These data support the conclusion that Mph1 contained two independent Fkh1-FHA binding motifs, each having a similar affinity for Fkh1.

### The Fkh1-Mph1 interaction contributed to the directionality of mating-type switching but not to *MPH1*’s role in tolerance for MMS-induced DNA damage or genome stability

**Fig 7:**
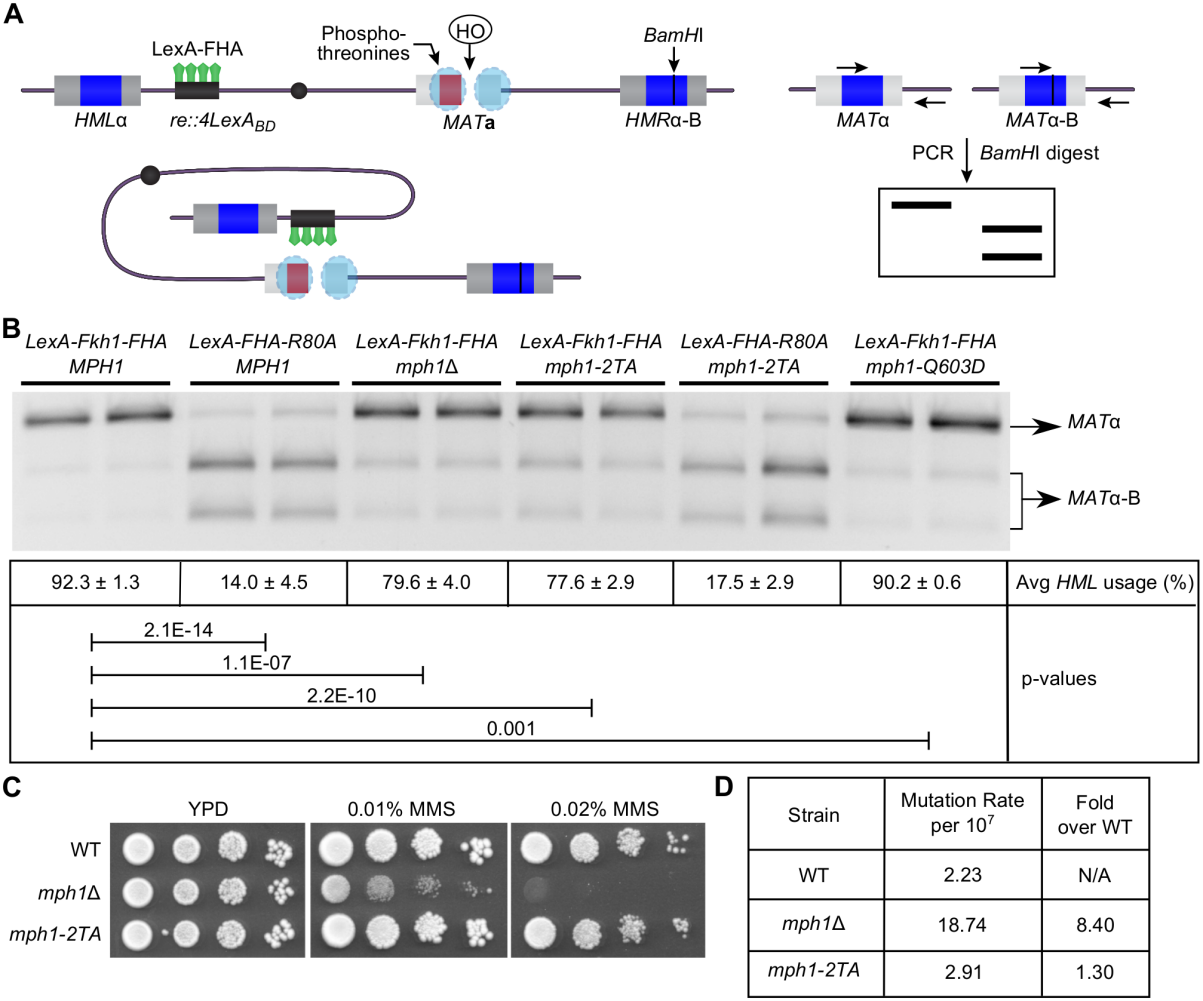
The Fkh1-Mph1 interaction contributed to the regulation of donor preference during mating-type switching. (**A**) Schematic showing Fkh1-dependent PCR-based switching assay and model proposed in [22]. A galactose-inducible HO endonuclease cuts at *MAT***a**. The resulting double strand break is repaired through recombination with either *HML* or *HMR*, both of which contain *MAT*α sequence, but with the insertion of a unique *BamH*I restriction site in *HMR* (*HMR*α-B). Directionality is controlled through the recombination enhancer (RE), which has been replaced by 4 LexA binding domains. LexA-fused Fkh1-FHA binds to RE causing a preference for recombination with *HML*. Primers that specifically amplify *MAT*α were used to amplify the repaired *MAT* locus by PCR. The PCR products were then digested with *BamH*I and donor preference was calculated as *MAT*α / (*MAT*α + *MAT*α-B). (**B**) A switching assay was performed using at least four replicates of each strain. Average *HML* usage and standard deviations were calculated and a representative gel is shown. Strains were congenic and contained all alleles represented in panel (A) unless otherwise noted. P-value significance of differences observed between strains is indicated by connecting lines. (**C**) MMS assays of *MPH1* mutant strains. Cells were grown in liquid YPD media to mid-log phase and 10-fold serial dilutions were spotted onto YPD plates containing the indicated concentration of MMS. (**D**) Mutation rate of *MPH1* mutant strains. Forward *CAN1* mutation rate was calculated using FALCOR by the Ma-Sandri-Sarkar maximum likelihood method in which the data are fit to the Luria-Delbrück distribution [53].

After establishing that the Fkh1-Mph1 interaction was mediated by the FHA domain of Fkh1 and one of two phosphothreonines on Mph1, we assessed whether this interaction was important for Fkh1’s role in mating-type switching. Fkh1 regulates donor preference during mating-type switching by directly binding to the recombination enhancer (RE) and promoting recombination between an HO-induced DSB at *MAT* and the donor locus *HML*. In a previous study, the N-terminal region of Fkh1 containing the FHA domain was shown to be sufficient to direct RE function [22]. This point was elucidated by engineering a strain in which RE was replaced with LexA binding sites and a LexA-Fkh1-FHA fusion protein was expressed [22]. In this Fkh1-dependent assay, the **a**-mating-type genes located at *HMR* were replaced by *MAT*α sequences that contained a unique *BamH*I restriction site (*HMR*α-B), such that repair of a DSB generated by the HO endonuclease at *MAT***a** will always result in a *MAT*α cell, and those using the *HMR*α-B donor sequence can be cut by *BamH*I, while those using *HML*α cannot. Thus donor preference can be examined by testing the relative abundance of the two different repair products through a PCR reaction that amplifies *MAT*α sequences followed by a *BamH*I restriction digest (Fig 7A). Consistent with a previous finding [22], *HML* was the preferred donor, as it was used as template for repair in >90% of cells, while in a strain containing a mutant version of LexA-FHA containing the R80A substitution (LexA-FHA-R80A), recombination between *MAT***a** and *HML* was reduced to less than 20% (Fig 7B). We found that *mph1*Δ reduced the function of RE, as *HML* now acted as the donor in <80% of cells (Fig 7B). While this level of reduction was not equivalent to that caused by loss of Fkh1-FHA function, it was highly reproducible. Moreover, *mph1-2TA* phenocopied the effect of the *mph1*Δ allele and reduced *HML* usage to <80%. Additionally, *mph1-2TA* did not reduce *HML* preference further in strains expressing LexA-FHA-R80A, providing additional genetic evidence that the Fkh1-Mph1 interaction contributed to donor preference during mating-type switching. The helicase activity of Mph1 is not responsible for this activity, as a helicase defective mutant of *MPH1 (mph1-Q603D*) did not alter donor preference as drastically as deletion of *MPH1* or the *mph1-2TA* allele, although it did have a statistically small effect. This donor preference defect caused by *mph1-2TA* was specific to this allele because, unlike *mph1*Δ cells, *mph1-2TA* cells did not exhibit sensitivity to MMS (Fig 7C) or an increase in mutation rate (Fig 7D). Thus the *mph1-2TA* allele caused a specific functional defect in Mph1’s role in regulating RE function while leaving at least two other known roles for Mph1 intact.

### *FDO1* also contributed to the regulation of donor preference during mating-type switching

**Fig 8:**
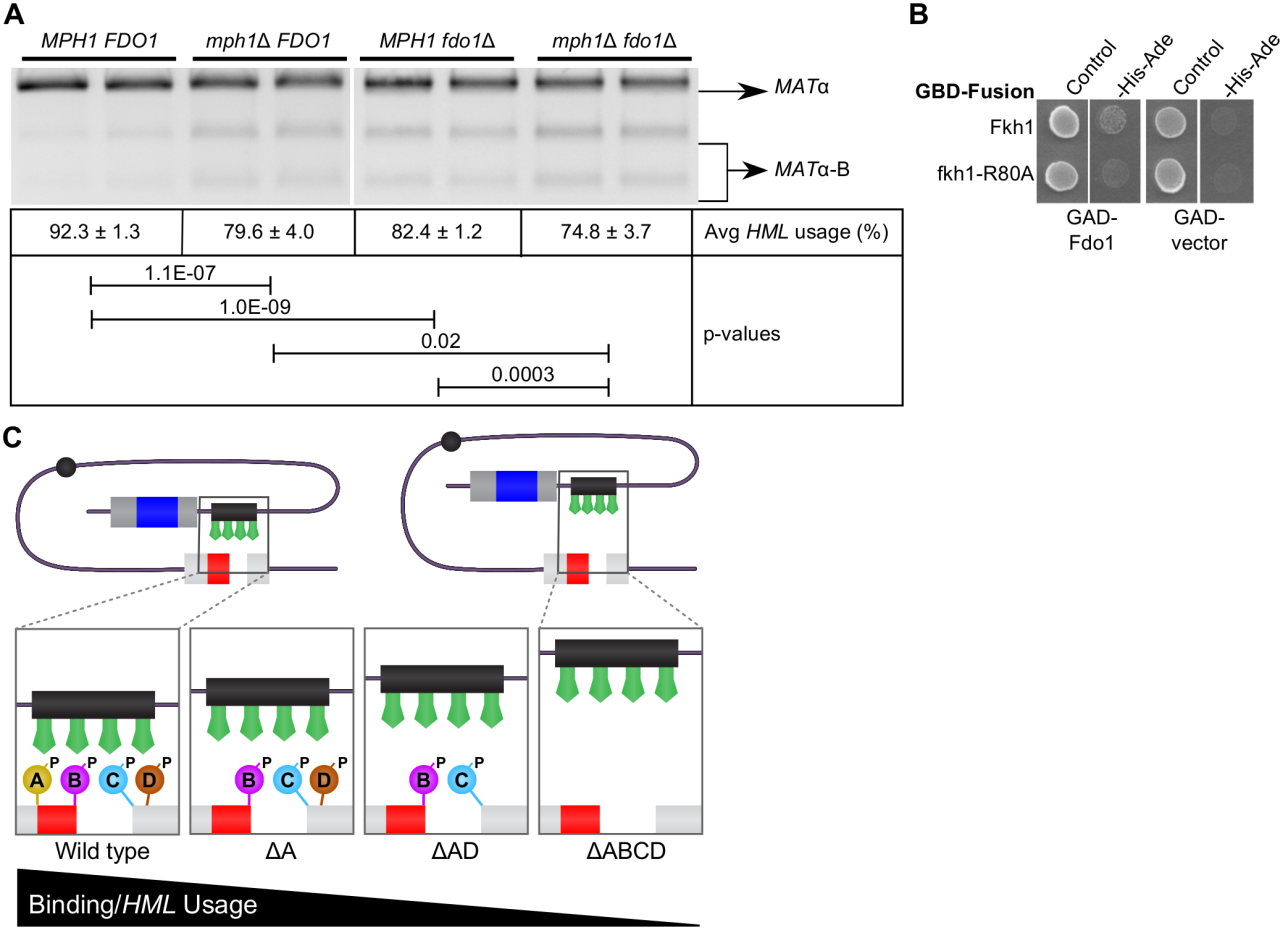
Fdo1 contributed to Fkh1-FHA-dependent regulation of donor preference during mating-type switching. (**A**) A switching assay was performed using at least four replicates of each strain. Average *HML* usage and standard deviations were calculated and a representative gel is shown. Strains were congenic and contained all alleles represented in Fig 7A unless otherwise noted. P-value significance of differences observed between strains is indicated by connecting lines. (**B**) Yeast 2-hybrid assays using different forms of full-length Fkh1 as bait. GAD constructs contained the region of Fdo1 identified in the 2-hybrid screen (Table 1) or the GAD alone. (**C**) Model for Fkh1 function at RE. Fkh1-FHA interacts with multiple proteins that associate with the DSB generated at *MAT* and phosphorylated on threonines (we represent only 4 putative phosphoproteins). Deletion of any single Fkh1-FHA partner only slightly reduces interaction with the DSB at MAT and, therefore, *HML* preference. Deletion of more than one partner reduces binding and *HML* preference further. Thus Fkh1-FHA’s role at the RE requires its interaction with many different proteins that together define a DSB.

The reduction in *HML* usage in *mph1-2TA* strains is less than that in cells expressing LexA-FHA-R80A, suggesting there must be other Fkh1 partners required for its role in mating-type switching. To address a role for additional Fkh1-FHA partner proteins, we examined the switching profile in cells lacking Fdo1. We found that deletion of *FDO1* reduces *HML* usage to ~80%, a 10% reduction relative to the wild type control similar to the level of reduction caused by deletion of *MPH1* (Fig 8A). Interestingly, in contrast to the Mph1-Fkh1 interaction, the Fkh1 FHA domain was not sufficient for interaction with Fdo1 (S2C Fig). However, further examination of this interaction by 2-hybrid showed that, in the context of full length Fkh1, the fkh1-R80A mutation reduced the Fkh1-Fdo1 interaction, strongly suggesting that the established phosphothreonine binding function of the FHA domain was necessary for the Fkh1-Fdo1 interaction as it was for the Fkh1-Mph1 interaction (Fig 8B). To test whether the defects in donor preference caused by deletions of *MPH1* and *FDO1* were additive, we also examined mating-type switching in *mph1*Δ *fdo1*Δ cells. *HML* usage was reduced in these cells to a greater degree than in cells containing either single mutation, suggesting that Mph1 and Fdo1 contribute independent Fkh1-FHA binding interactions to control Fkh1-regulated donor preference.

## Discussion

This study provided evidence that Mph1 was a direct Fkh1-FHA phosphoprotein partner relevant to Fkh1’s role in regulating the directionality of mating-type switching. This Fkh1-Mph1 interaction was mediated through a small peptide within the C-terminal regulatory region of Mph1 that contains two threonines each capable of directing interactions with the Fkh1 FHA domain. Mutagenesis studies show that these two threonines likely act as two independent Fkh1-FHA binding motifs, as both threonines must be substituted with alanine to abolish binding by 2-hybrid. Additionally, the amino acid sequences surrounding the two threonines are similar and highly acidic. Both motifs have a glutamic acid residue at the pT+1 position, and mutational analyses indicated that this residue was important for each motif to direct binding of the Fkh1 FHA domain to Mph1. While the 2-hybrid data cannot exclude the possibility that the +1 glutamic acid is required for phosphorylation of the relevant threonine and not directly involved in Fkh1-FHA binding, they nevertheless indicate that a TE signature is relevant to each motif’s independent ability to direct an Mph1-Fkh1-FHA interaction. These observations underscore that there are two redundant Fkh1-FHA binding motifs built into this small region of Mph1. Because a mutant incapable of phosphorylation on these threonines, *mph1-2TA*, behaved as an *mph1*Δ in a mating-type switching assay, but not in other commonly used assays that assess *MPH1* function, we propose that the Fkh1-Mph1 interaction helps establish the long-range chromosomal interaction essential for donor preference during mating-type switching.

While our data were consistent with the model for Fkh1 bound to the recombination enhancer (RE) guiding the *HML* locus to the DSB at *MAT* [22], they also raised an important new question. In particular, why does loss of Fkh1-FHA function cause a much larger defect in RE function compared to *mph1-2TA* (or *mph1*Δ), both of which abolish Fkh1-FHA-Mph1 interactions? The simplest explanation is that Mph1 is only one of several proteins bound to the DSB at *MAT* that the Fkh1 FHA domain uses to locate this lesion. It makes sense for Fkh1 to bind several different proteins at the DSB with relatively weak affinities—in this way the RE remains close to *MAT* long enough to increase the opportunity for strand invasion into *HML*. At the same time Fkh1 is not bound so tightly to any one partner or the DSB region itself to inhibit strand invasion and the protein/DNA remodeling necessary to drive the recombination event. Therefore, we propose that there must exist other Fkh1-FHA partner proteins at the HO-induced DSB at *MAT* that contribute to the RE’s ability to direct the *MAT* locus to *HML*. The multi-partner model for Fkh1 FHA function in donor preference may represent a general mechanism by which Fkh1 FHA performs its other biological functions in transcription and replication. This type of mechanism may allow for relatively high specificity but low affinity (and thus potentially highly dynamic) interactions that may be important to these complex chromosomal processes.

Based on this idea and data reported in a previous study, the CK2 kinase likely phosphorylates many Fkh1-interacting proteins involved in donor preference [22]. In this regard we note that, consistent with our observation of an interaction in asynchronous cells and within the 2-hybrid context, CK2 constitutively phosphorylates target proteins [54]. Additionally, the amino acid sequence surrounding both relevant Mph1 threonines are consistent with a CK2 target [54]. When these phosphorylated proteins come together at a DSB, perhaps with other proteins phosphorylated in a more regulated manner by other kinases, they collectively serve to define the DSB for Fkh1-FHA. Consistent with this proposal, a deletion of *FDO1*, a gene encoding another Fkh1-FHA interaction partner identified in our screen, also reduced donor preference to a degree similar to that of *mph1-2TA* (or *mph1*Δ). Moreover, a deletion of both genes to create an *fdo1*Δ *mph1*Δ cell reduced preference for *HML* to a degree greater than deletion of either gene alone. However, a substantial amount of Fkh1-FHA-dependent donor preference remained intact even in cells carrying null mutations in both of these genes, suggesting that another protein or proteins at the DSB must interact with Fkh1-FHA. Many proteins, in addition to Mph1, bind to DSBs and would be good candidates for additional Fkh1-FHA interaction partners that regulate donor preference [55–57]. While mating-type switching is a specific form of homologous recombination, it is clear that DSB repair in diploids also requires a search for homologous regions by the DSB [58]. It will be interesting to learn whether this more generalized process uses similar protein-protein interactions to stabilize chromosomal interactions that serve to juxtapose homologous regions.

Our data provided evidence that the Fkh1 FHA domain may be controlling most, if not all, Fkh1-mediated biology in yeast. Indeed, many *fkh1-fha* single residue substitution (*fkh1-m*) mutants abolished interaction with all protein partners uncovered here and reduced Fkh1’s ability to function in cell-cycle regulation with Fkh2, even though deletion of no single gene encoding an interaction partner had an effect. Based on the results with donor preference, it seems likely that multiple different Fkh1-FHA interaction partners will be needed to fully explain Fkh1-FHA’s role in cell cycle regulation. A deletion of the entire FHA domain of Fkh1 (*fkh1-fha*Δ) phenocopied a *fkh1*Δ mutation in cell cycle regulation as measured by both mitotic cell division rates and pseudohyphal-like growth and agar scarring when combined with a *fkh2*Δ allele. Because the established role of FHA domains is to bind phosphopeptides, it was perhaps unexpected that amino acid substitutions in the FHA domain predicted to abolish FHA-phosphopeptide interactions only slowed mitotic cell division in *fkh2*Δ cells without causing pseudohyphal-like growth. The Fkh1 FHA domain may play roles in Fkh1 function in addition to phosphopeptide binding by providing as yet undefined interaction surfaces for other regulators of transcription. Alternatively, the *fkh1-fha*Δ allele used in this study lacked coding information for an additional ~30 amino acids outside of the alignment-defined FHA domain that may provide surfaces for additional protein-protein interactions. Regardless, these data raise new questions about whether Fkh1’s roles in regulating cell proliferation rate and suppressing pseudohyphal growth are completely separable, or whether a certain threshold of reduced transcription/altered transcriptional regulation must be met before pseudohyphal growth is also observed.

Our data provided evidence that several Fkh1-FHA interaction partners that can direct Fkh1 cellular roles remain unidentified. As we have shown, determining the role of any particular Fkh1-protein interaction is difficult through mutation of Fkh1-FHA itself, as the same FHA residues participate in multiple Fkh1-protein interactions and Fkh1 processes. For this reason, it will be important to identify other Fkh1-FHA-partner proteins and engineer mutations that specifically abolish their ability to interact with Fkh1, as we did for Mph1 in this study, to isolate the discrete mechanisms and pathways influenced by Fkh1.

## Materials and Methods

### Strains and plasmids

Strains used in this study were derived from the *Saccharomyces cerevisiae* strain w303 unless otherwise noted. Standard methods were used for yeast growth, strain and plasmid construction. Strains used in this study are listed in S1 Table. Plasmids are listed in S2 Table. Random mutagenesis of pGBDU-C1 plasmids was performed as described in [59]. Lack of interaction alleles were identified by replica plating from non-selective media to media selective for 2-hybrid interaction and identifying colonies that were no longer viable. Mutants identified by random mutagenesis were confirmed by directed mutagenesis and 2-hybrid assays.

### Yeast 2-hybrid assays

2-hybrid assays were performed in the PJ69-4A strain as described in [29]. The strain contains two reporter genes, *HIS3* and *ADE2*. The original screen was performed using a Fkh1-GBD fusion protein in which the entire DNA binding domain was precisely replaced with the GBD. This GBD-Fkh1 fusion activated transcription of the *HIS3* reporter gene. Therefore colonies harboring potential Fkh1-interacting partners were identified on minimal media lacking both histidine and adenine.

### Homology modeling and FHA alignment

A predicted structure for the Fkh1 FHA domain was generated using the N-terminal Rad53 FHA domain as a template using SWISS-MODEL [42–45]. Amino acids 72–170 were modeled. A structure-based sequence alignment of the N-terminal Rad53 FHA domain (Rad53-1) and the Fkh1 FHA domain was generated using a combination of the Rad53 crystal structure (PDB 1G6G) [42] and structural predictions of the Fkh1 FHA domain based on a combination of the homology model and secondary structure predicted using JPred [46]. Electrostatic potential was generated by PyMol v 1.7 [60].

### Determining morphology and growth rates

Heterozygous *fkh1-m*/+ *fkh2*Δ/+ diploids expressing Fkh1 mutants were dissected and scanned after three days growth. Agar scarring was assessed by gently patching haploid strains onto YPD and washing with H_2_O after three days. Growth curves were generated by growing to saturation in YPD media, diluting to an OD_600_ of 0.1 in a 96-well plate, and monitoring growth by measuring the OD_600_ every three minutes over a 24 hour period in a Biotek Synergy 2 plate reader shaking at 30°C. Doubling times were calculated by exponential regression of data generated from growth curves during log-phase [61].

### Co-immunoprecipitation and western blotting

Cell extracts for western blotting were prepared as described in [62]. Cell extracts for co-immunoprecipitation were prepared by breaking cells by the glass bead method in CoIP buffer (50 mM HEPES pH 7.5, 140 mM NaCl, 1 mM EDTA, 1% TX-100, protease inhibitors (Calbiotech)). Lysates were then diluted 1:1 in CoIP buffer and incubated with the appropriate antibody. Beads were washed with CoIP buffer without detergents followed by washes with the same buffer with 200 mM NaCl.

Co-immunoprecipitation of Fkh1 and FLAG-tagged Mph1 (modified from [63]) were performed using Anti-FLAG antibodies (ANTI-FLAG M2 Affinity Gel, Sigma) or Protein A sepharose-linked anti-Fkh1 antibodies [64]. The starting extract and immunoprecipitated proteins were examined by protein immunoblotting using either anti-FLAG (ANTI-FLAG M2 monoclonal, Sigma) or anti-Fkh1 antibodies. Orc1 detected with an anti-Orc1 antibody [49] served as a loading control.

### Fluorescence anisotropy

C-terminally His-tagged full length Fkh1 protein was expressed from a pET28b expression vector in Rosetta *E*. *coli*. *E*. *coli* were broken with modified B-PER (Thermo Fisher) diluted 1:1 in wash buffer (50 mM Tris pH 7.0, 5 mM MgCl_2_, 5 mM ATP, 10% glycerol, 1M NaCl, 5 mM BME, 20 mM imidazole, protease inhibitors (Calbiotech)) with 1 mM EDTA. His-tagged Fkh1 protein was purified using nickel chromatography (Qiagen) and eluted in buffer (Wash buffer with 200 mM NaCl, 500 mM imidazole, and without ATP). Peptides (synthesized by the University of Wisconsin-Madison and the Tufts University Core Facility) were labeled on the N-terminus with 5-carboxy fluorescein and an aminohexanoic acid linker. Peptides (constant final concentration of 3 nM) were mixed with titrations of purified Fkh1-6xHis protein in binding buffer (50 mM HEPES pH 7.0, 200 mM KCl, 10% glycerol, 5 mM BME, 1 mM EDTA, 1 mM EGTA, 5 mM MgOAc, 0.02% NP-40, protease inhibitors (Calbiotech)). Polarization at each concentration was measured in triplicates in 384-well polystyrene black microplates (Thermo Fisher Scientific #262260) by a Biotek Synergy H4 multimode plate reader (light source: xenon flash, offset from top: 7 mm, sensitivity: 60%, excitation: 485/20 nm, emission: 528/20 nm, both parallel and perpendicular, normal read speed). Fraction bound (Fb) at each concentration was calculated based on the corresponding polarization values (P): Fb_c_ = (P_c_—P_min_) / (P_max_—P_min_), where P_min_ is the polarization value of the no-protein control and P_max_ is the polarization value of the saturation value for that peptide. Dissociation constants (K_d_) were derived by KaleidaGraph (version 4.1.3) using the following equation:

$$\text{Fb }=\;\left[\text{protein}\right]\;/\;(\left[\text{protein}\right]\;+\;{\text{K}}_{\text{d}})$$

### Mutation rate analysis and MMS assays

Mutation rates were calculated by fluctuation analysis as in [65]. Briefly, single colonies were inoculated into minimal media lacking arginine and grown overnight, diluted 1:10,000 and aliquoted into a 96-well plate. Cells were then incubated, without shaking, at 30°C for 2 days. 24 of the 96 samples were pooled and plated in triplicate to determine the number of viable cells. The remaining 72 samples were spotted onto 10x canavanine plates (minimal media lacking arginine + 0.6 g/L canavanine). Mutation rate was analyzed using FALCOR by the Ma-Sandri-Sarkar maximum likelihood method in which the data are fit to the Luria-Delbrück distribution [53]. For MMS assays, cells were grown to mid-log phase, diluted so that the OD_600_ is 0.5 and 10-fold serial dilutions were spotted onto YPD plates containing the indicated concentration of MMS. MMS plates were poured fresh on the day of each experiment. Plates were imaged three days after plating.

### Mating-type switching assays

Donor preference during mating-type switching was determined by a PCR-based method as described in [22]. Briefly, cells were grown in YP-lactate medium to mid-log phase. Expression of the HO endonuclease was induced by addition of 2% galactose and incubated for one hour. Induction was stopped by the addition of 2% glucose and the cells were allowed to recover for 24 hours. DNA was then isolated using quick genomic DNA extraction [66] and PCR was used to amplify *MAT*α sequences using primers Yalpha105F and MAT-dist4R [22]. 700 ng of PCR DNA was then cut with *BamH*I and the resulting digest was run on an agarose gel. Relative densities of the different bands were determined using ImageJ [67], and donor preference (as *HML* usage) was calculated using the formula *MAT*α / (*MAT*α+*MAT*α-B).

## Supporting Information

S1 Fig Homology modeling of Fkh1 FHA domain. (**A**) QMEAN4 scores of homology models generated of the Fkh1 FHA domain. QMEAN4 scores provided by SWISS-MODEL [68]. (**B**) Predicted residue error of every amino acid residue (in Ångström) in the Fkh1 homology model as assessed by the QMEAN scoring function. Provided by SWISS-MODEL [68].
(TIF)

S2 Fig Additional yeast 2-hybrid analyses of Fkh1 mutants. (**A-C**) Yeast 2-hybrid assays using different mutant forms of Fkh1 bait. The FHA domain is defined as amino acids 50–202. GAD constructs contain the segment of each protein identified in the original 2-hybrid screen (listed in Table 1) or the GAD alone.
(TIF)

S3 Fig Model of electrostatic potential of the Fkh1 FHA domain. (**A**) Model of electrostatic potential. Blue indicates positively charged regions. Red indicates negatively charged regions. (**B**) The positively charged region on the phosphopeptide interaction surface contains residues R80, K107, R111, K112, and R132. See methods section for details on the generation of the Fkh1 structure model.
(TIF)

S1 Table Yeast strains used in this study. (DOCX)

S2 Table Plasmids used in this study. (DOCX)

## References

[1] Kaufmann E, Knöchel W. Five years on the wings of fork head. Mech Dev. 1996;57: 3–20. 8817449 10.1016/0925-4773(96)00539-4

[2] Spellman PT, Sherlock G, Zhang MQ, Iyer VR, Anders K, Eisen MB, et al Comprehensive identification of cell cycle-regulated genes of the yeast *Saccharomyces cerevisiae* by microarray hybridization. Mol Biol Cell. 1998;9: 3273–3297. 9843569 10.1091/mbc.9.12.3273 PMC25624

[3] Hollenhorst PC, Bose ME, Mielke MR, Müller U, Fox CA. Forkhead genes in transcriptional silencing, cell morphology and the cell cycle. Overlapping and distinct functions for *FKH1* and *FKH2* in *Saccharomyces cerevisiae*. Genetics. 2000;154: 1533–1548. 10747051 10.1093/genetics/154.4.1533 PMC1461039

[4] Koranda M, Schleiffer A, Endler L, Ammerer G. Forkhead-like transcription factors recruit Ndd1 to the chromatin of G2/M-specific promoters. Nature. 2000;406: 94–98. 10.1038/35017589 10894549

[5] Kumar R, Reynolds DM, Shevchenko A, Shevchenko A, Goldstone SD, Dalton S. Forkhead transcription factors, Fkh1p and Fkh2p, collaborate with Mcm1p to control transcription required for M-phase. Curr Biol. 2000;10: 896–906. 10959837 10.1016/s0960-9822(00)00618-7

[6] Pic A, Lim FL, Ross SJ, Veal EA, Johnson AL, Sultan MR, et al The forkhead protein Fkh2 is a component of the yeast cell cycle transcription factor SFF. EMBO J. 2000;19: 3750–3761. 10.1093/emboj/19.14.3750 10899128 PMC313965

[7] Zhu G, Spellman PT, Volpe T, Brown PO, Botstein D, Davis TN, et al Two yeast forkhead genes regulate the cell cycle and pseudohyphal growth. Nature. 2000;406: 90–94. 10.1038/35017581 10894548

[8] Boros J, Lim F-L, Darieva Z, Pic-Taylor A, Harman R, Morgan BA, et al Molecular determinants of the cell-cycle regulated Mcm1p-Fkh2p transcription factor complex. Nucleic Acids Res. 2003;31: 2279–2288. 12711672 10.1093/nar/gkg347 PMC154233

[9] Darieva Z, Pic-Taylor A, Boros J, Spanos A, Geymonat M, Reece RJ, et al Cell cycle-regulated transcription through the FHA domain of Fkh2p and the coactivator Ndd1p. Curr Biol. 2003;13: 1740–1745. 14521842 10.1016/j.cub.2003.08.053

[10] Reynolds D, Shi BJ, McLean C, Katsis F, Kemp B, Dalton S. Recruitment of Thr 319-phosphorylated Ndd1p to the FHA domain of Fkh2p requires Clb kinase activity: a mechanism for *CLB* cluster gene activation. Genes Dev. 2003;17: 1789–1802. 10.1101/gad.1074103 12865300 PMC196186

[11] Pic-Taylor A, Darieva Z, Morgan BA, Sharrocks AD. Regulation of cell cycle-specific gene expression through cyclin-dependent kinase-mediated phosphorylation of the forkhead transcription factor Fkh2p. Mol Cell Biol. 2004;24: 10036–10046. 10.1128/MCB.24.22.10036-10046.2004 15509804 PMC525469

[12] Veis J, Klug H, Koranda M, Ammerer G. Activation of the G2/M-Specific Gene *CLB2* Requires Multiple Cell Cycle Signals. Mol Cell Biol. 2007;27: 8364–8373. 10.1128/MCB.01253-07 17908798 PMC2169163

[13] Hollenhorst PC, Pietz G, Fox CA. Mechanisms controlling differential promoter-occupancy by the yeast forkhead proteins Fkh1p and Fkh2p: implications for regulating the cell cycle and differentiation. Genes Dev. 2001;15: 2445–2456. 10.1101/gad.906201 11562353 PMC312786

[14] Knott SRV, Peace JM, Ostrow AZ, Gan Y, Rex AE, Viggiani CJ, et al Forkhead transcription factors establish origin timing and long-range clustering in *S*. *cerevisiae*. Cell. 2012;148: 99–111. 10.1016/j.cell.2011.12.012 22265405 PMC3266545

[15] Lõoke M, Kristjuhan K, Värv S, Kristjuhan A. Chromatin-dependent and -independent regulation of DNA replication origin activation in budding yeast. EMBO Rep. 2012;14: 191–198. 10.1038/embor.2012.196 23222539 PMC3596130

[16] Sun K, Coïc E, Zhou Z, Durrens P, Haber JE. *Saccharomyces* forkhead protein Fkh1 regulates donor preference during mating-type switching through the recombination enhancer. Genes Dev. 2002;16: 2085–2096. 10.1101/gad.994902 12183363 PMC186439

[17] Coïc E, Sun K, Wu C, Haber JE. Cell cycle-dependent regulation of *Saccharomyces cerevisiae* donor preference during mating-type switching by SBF (Swi4/Swi6) and Fkh1. Mol Cell Biol. 2006;26: 5470–5480. 10.1128/MCB.02443-05 16809780 PMC1592702

[18] Strathern JN, Herskowitz I. Asymmetry and directionality in production of new cell types during clonal growth: the switching pattern of homothallic yeast. Cell. 1979;17: 371–381. 378408 10.1016/0092-8674(79)90163-6

[19] Haber JE. Mating-type genes and *MAT* switching in *Saccharomyces cerevisiae*. Genetics. 2012;191: 33–64. 10.1534/genetics.111.134577 22555442 PMC3338269

[20] Haber JE. Mating-type gene switching in *Saccharomyces cerevisiae*. Annu Rev Genet. 1998;32: 561–599. 10.1146/annurev.genet.32.1.561 9928492

[21] Wu X, Haber JE. A 700 bp cis-acting region controls mating-type dependent recombination along the entire left arm of yeast chromosome III. Cell. 1996;87: 277–285. 8861911 10.1016/s0092-8674(00)81345-8

[22] Li J, Coïc E, Lee K, Lee C-S, Kim J-A, Wu Q, et al Regulation of budding yeast mating-type switching donor preference by the FHA domain of Fkh1. PLoS Genet. 2012;8: e1002630 10.1371/journal.pgen.1002630 22496671 PMC3320585

[23] Ercan S, Reese JC, Workman JL, Simpson RT. Yeast Recombination Enhancer Is Stimulated by Transcription Activation. Mol Cell Biol. 2005;25: 7976–7987. 10.1128/MCB.25.18.7976-7987.2005 16135790 PMC1234320

[24] Durocher D, Henckel J, Fersht AR, Jackson SP. The FHA domain is a modular phosphopeptide recognition motif. Mol Cell. 1999;4: 387–394. 10518219 10.1016/s1097-2765(00)80340-8

[25] Durocher D, Jackson SP. The FHA domain. FEBS Lett. 2002;513: 58–66. 10.1016/S0014-5793(01)03294-X 11911881

[26] Mohammad DH, Yaffe MB. 14-3-3 proteins, FHA domains and BRCT domains in the DNA damage response. DNA Repair. 2009;8: 1009–1017. 10.1016/j.dnarep.2009.04.004 19481982 PMC3263375

[27] Mermershtain I, Glover JNM. Structural mechanisms underlying signaling in the cellular response to DNA double strand breaks. Mutat Res. 2013;750: 15–22. 10.1016/j.mrfmmm.2013.07.004 23896398 PMC3818410

[28] Reinhardt HC, Yaffe MB. Phospho-Ser/Thr-binding domains: navigating the cell cycle and DNA damage response. Nat Rev Mol Cell Biol. 2013;14: 563–580. 10.1038/nrm3640 23969844

[29] James P, Halladay J, Craig EA. Genomic libraries and a host strain designed for highly efficient two-hybrid selection in yeast. Genetics. 1996;144: 1425–1436. 8978031 10.1093/genetics/144.4.1425 PMC1207695

[30] Scheller J, Schürer A, Rudolph C, Hettwer S, Kramer W. *MPH1*, a yeast gene encoding a DEAH protein, plays a role in protection of the genome from spontaneous and chemically induced damage. Genetics. 2000;155: 1069–1081. 10880470 10.1093/genetics/155.3.1069 PMC1461162

[31] Mitchell AP, Magasanik B. Regulation of glutamine-repressible gene products by the *GLN3* function in Saccharomyces cerevisiae. Mol Cell Biol. 1984;4: 2758–2766. 6152012 10.1128/mcb.4.12.2758-2766.1984 PMC369286

[32] Courchesne WE, Magasanik B. Regulation of nitrogen assimilation in Saccharomyces cerevisiae: roles of the *URE2* and *GLN3* genes. J Bacteriol. 1988;170: 708–713. 2892826 10.1128/jb.170.2.708-713.1988 PMC210712

[33] Lussier M, White AM, Sheraton J, di Paolo T, Treadwell J, Southard SB, et al Large scale identification of genes involved in cell surface biosynthesis and architecture in *Saccharomyces cerevisiae*. Genetics. 1997;147: 435–450. 9335584 10.1093/genetics/147.2.435 PMC1208169

[34] Giaever G, Chu AM, Ni L, Connelly C, Riles L, Véronneau S, et al Functional profiling of the *Saccharomyces cerevisiae* genome. Nature. 2002;418: 387–391. 10.1038/nature00935 12140549

[35] Ho Y, Gruhler A, Heilbut A, Bader GD, Moore L, Adams S-L, et al Systematic identification of protein complexes in *Saccharomyces cerevisiae* by mass spectrometry. Nature. 2002;415: 180–183. 10.1038/415180a 11805837

[36] Whitby MC. The FANCM family of DNA helicases/translocases. DNA Repair. 2010;9: 224–236. 10.1016/j.dnarep.2009.12.012 20117061

[37] Xue X, Sung P, Zhao X. Functions and regulation of the multitasking FANCM family of DNA motor proteins. Genes Dev. 2015;29: 1777–1788. 10.1101/gad.266593.115 26341555 PMC4573851

[38] Banerjee S, Smith S, Oum J-H, Liaw H-J, Hwang J-Y, Sikdar N, et al Mph1p promotes gross chromosomal rearrangement through partial inhibition of homologous recombination. J Cell Biol. 2008;181: 1083–1093. 10.1083/jcb.200711146 18591428 PMC2442200

[39] Chavez A, Agrawal V, Johnson FB. Homologous recombination-dependent rescue of deficiency in the structural maintenance of chromosomes (Smc) 5/6 complex. J Biol Chem. 2011;286: 5119–5125. 10.1074/jbc.M110.201608 21138837 PMC3037623

[40] Xue X, Choi K, Bonner J, Chiba T, Kwon Y, Xu Y, et al Restriction of Replication Fork Regression Activities by a Conserved SMC Complex. Mol Cell. 2014;56: 436–445. 10.1016/j.molcel.2014.09.013 25439736 PMC4268010

[41] Xue X, Choi K, Bonner JN, Szakal B, Chen Y-H, Papusha A, et al Selective modulation of the functions of a conserved DNA motor by a histone fold complex. Genes Dev. 2015;29: 1000–1005. 10.1101/gad.259143.115 25956905 PMC4441048

[42] Durocher D, Taylor IA, Sarbassova D, Haire LF, Westcott SL, Jackson SP, et al The molecular basis of FHA domain:phosphopeptide binding specificity and implications for phospho-dependent signaling mechanisms. Mol Cell. 2000;6: 1169–1182. 11106755 10.1016/s1097-2765(00)00114-3

[43] Arnold K, Bordoli L, Kopp J, Schwede T. The SWISS-MODEL workspace: a web-based environment for protein structure homology modelling. Bioinformatics. 2006;22: 195–201. 10.1093/bioinformatics/bti770 16301204

[44] Kiefer F, Arnold K, Künzli M, Bordoli L, Schwede T. The SWISS-MODEL Repository and associated resources. Nucleic Acids Res. 2009;37: D387–392. 10.1093/nar/gkn750 18931379 PMC2686475

[45] Guex N, Peitsch MC, Schwede T. Automated comparative protein structure modeling with SWISS-MODEL and Swiss-PdbViewer: a historical perspective. Electrophoresis. 2009;30 Suppl 1: S162–173. 10.1002/elps.200900140 19517507

[46] Cole C, Barber JD, Barton GJ. The Jpred 3 secondary structure prediction server. Nucleic Acids Res. 2008;36: W197–201. 10.1093/nar/gkn238 18463136 PMC2447793

[47] Huang YM, Chang CA. Achieving peptide binding specificity and promiscuity by loops: Case of the Forkhead-Associated Domain. PLoS ONE. 2014;9: e98291 10.1371/journal.pone.0098291 24870410 PMC4037201

[48] Li J, Lee GI, Van Doren SR, Walker JC. The FHA domain mediates phosphoprotein interactions. J Cell Sci. 2000;113 Pt 23: 4143–4149. 11069759 10.1242/jcs.113.23.4143

[49] Gabrielse C, Miller CT, McConnell KH, DeWard A, Fox CA, Weinreich M. A Dbf4p BRCA1 C-terminal-like domain required for the response to replication fork arrest in budding yeast. Genetics. 2006;173: 541–555. 10.1534/genetics.106.057521 16547092 PMC1526507

[50] Schürer KA, Rudolph C, Ulrich HD, Kramer W. Yeast *MPH1* gene functions in an error-free DNA damage bypass pathway that requires genes from Homologous recombination, but not from postreplicative repair. Genetics. 2004;166: 1673–1686. 15126389 10.1534/genetics.166.4.1673 PMC1470801

[51] Nishino T, Komori K, Tsuchiya D, Ishino Y, Morikawa K. Crystal structure and functional implications of *Pyrococcus furiosus* hef helicase domain involved in branched DNA processing. Structure. 2005;13: 143–153. 10.1016/j.str.2004.11.008 15642269

[52] Liang X, Van Doren SR. Mechanistic Insights into Phosphoprotein-Binding FHA Domains. Acc Chem Res. 2008;41: 991–999. 10.1021/ar700148u 18656966 PMC2962622

[53] Hall BM, Ma C-X, Liang P, Singh KK. Fluctuation analysis CalculatOR: a web tool for the determination of mutation rate using Luria-Delbruck fluctuation analysis. Bioinformatics. 2009;25: 1564–1565. 10.1093/bioinformatics/btp253 19369502 PMC2687991

[54] Meggio F, Pinna LA. One-thousand-and-one substrates of protein kinase CK2? FASEB J. 2003;17: 349–368. 10.1096/fj.02-0473rev 12631575

[55] Prakash R, Satory D, Dray E, Papusha A, Scheller J, Kramer W, et al Yeast Mph1 helicase dissociates Rad51-made D-loops: implications for crossover control in mitotic recombination. Genes Dev. 2009;23: 67–79. 10.1101/gad.1737809 19136626 PMC2632165

[56] Polo SE, Jackson SP. Dynamics of DNA damage response proteins at DNA breaks: a focus on protein modifications. Genes Dev. 2011;25: 409–433. 10.1101/gad.2021311 21363960 PMC3049283

[57] Krejci L, Altmannova V, Spirek M, Zhao X. Homologous recombination and its regulation. Nucleic Acids Res. 2012;40: 5795–5818. 10.1093/nar/gks270 22467216 PMC3401455

[58] Miné-Hattab J, Rothstein R. Increased chromosome mobility facilitates homology search during recombination. Nat Cell Biol. 2012;14: 510–517. 10.1038/ncb2472 22484485

[59] Muhlrad D, Hunter R, Parker R. A rapid method for localized mutagenesis of yeast genes. Yeast. 1992;8: 79–82. 10.1002/yea.320080202 1561838

[60] The PyMOL Molecular Graphics System. Schrödinger, LLC;

[61] Roth V. Doubling Time [Internet]. 2006. Available: http://www.doubling-time.com/compute.php

[62] Zhang T, Lei J, Yang H, Xu K, Wang R, Zhang Z. An improved method for whole protein extraction from yeast *Saccharomyces cerevisiae*. Yeast. 2011;28: 795–798. 10.1002/yea.1905 21972073

[63] Chen Y-H, Choi K, Szakal B, Arenz J, Duan X, Ye H, et al Interplay between the Smc5/6 complex and the Mph1 helicase in recombinational repair. Proc Natl Acad Sci. 2009;106: 21252–21257. 10.1073/pnas.0908258106 19995966 PMC2795505

[64] Casey L, Patterson EE, Müller U, Fox CA. Conversion of a replication origin to a silencer through a pathway shared by a forkhead transcription factor and an S phase cyclin. Mol Biol Cell. 2008;19: 608–622. 10.1091/mbc.E07-04-0323 18045995 PMC2230585

[65] Lang GI, Murray AW. Estimating the per-base-pair mutation rate in the yeast *Saccharomyces cerevisiae*. Genetics. 2008;178: 67–82. 10.1534/genetics.107.071506 18202359 PMC2206112

[66] Lõoke M, Kristjuhan K, Kristjuhan A. Extraction of genomic DNA from yeasts for PCR-based applications. BioTechniques. 2011;50: 325–328. 10.2144/000113672 21548894 PMC3182553

[67] Schneider CA, Rasband WS, Eliceiri KW. NIH Image to ImageJ: 25 years of image analysis. Nat Methods. 2012;9: 671–675. 10.1038/nmeth.2089 22930834 PMC5554542

[68] Benkert P, Biasini M, Schwede T. Toward the estimation of the absolute quality of individual protein structure models. Bioinformatics. 2011;27: 343–350. 10.1093/bioinformatics/btq662 21134891 PMC3031035

