# Supplementary figures and images for: Binding of the Fkh1 Forkhead Associated Domain to a Phosphopeptide within the Mph1 DNA Helicase Regulates Mating-Type Switching in Budding Yeast
Source: PLoS Genet. 2016 Jun 3;12(6):e1006094. doi: 10.1371/journal.pgen.1006094 (PMC4892509; doi:10.1371/journal.pgen.1006094)

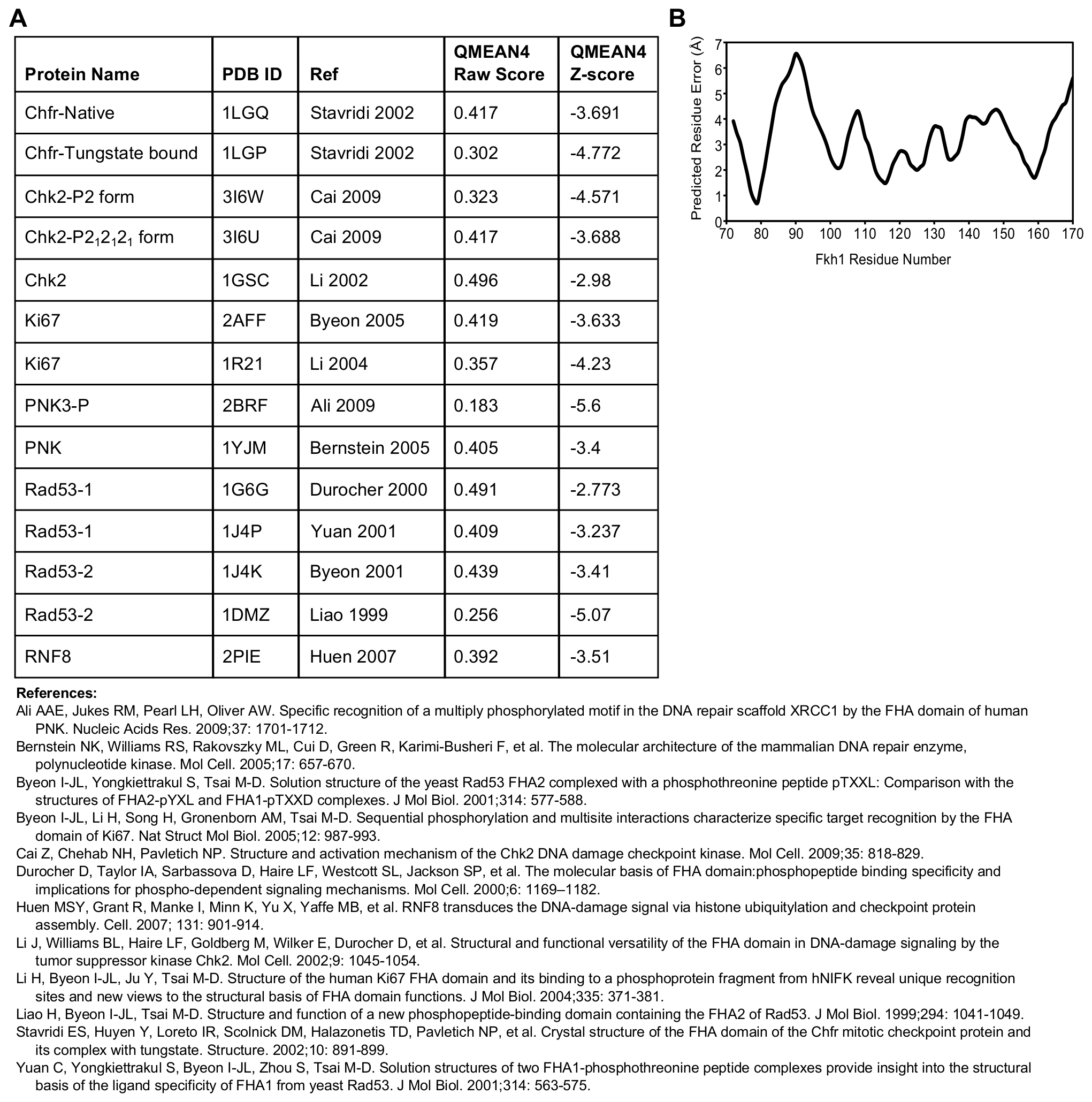

Supplement: S1 Fig — (A) QMEAN4 scores of homology models generated of the Fkh1 FHA domain. QMEAN4 scores provided by SWISS-MODEL [68]. (B) Predicted residue error of every amino acid residue (in Ångström) in the Fkh1 homology model as assessed by the QMEAN scoring function. Provided by SWISS-MODEL [68]. (TIF) [file pgen.1006094.s001.tif]

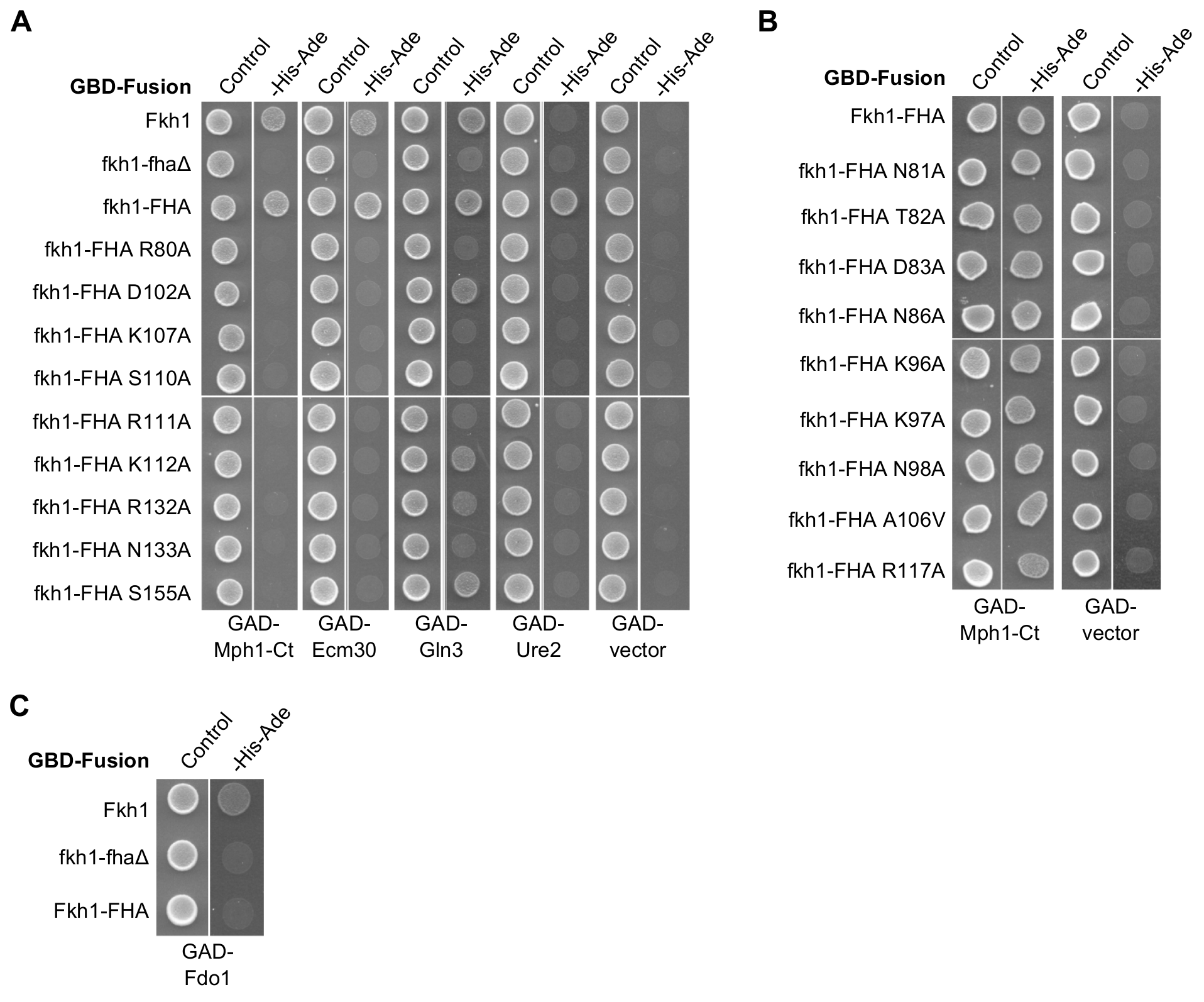

Supplement: S2 Fig — (A-C) Yeast 2-hybrid assays using different mutant forms of Fkh1 bait. The FHA domain is defined as amino acids 50–202. GAD constructs contain the segment of each protein identified in the original 2-hybrid screen (listed in Table 1) or the GAD alone. (TIF) [file pgen.1006094.s002.tif]

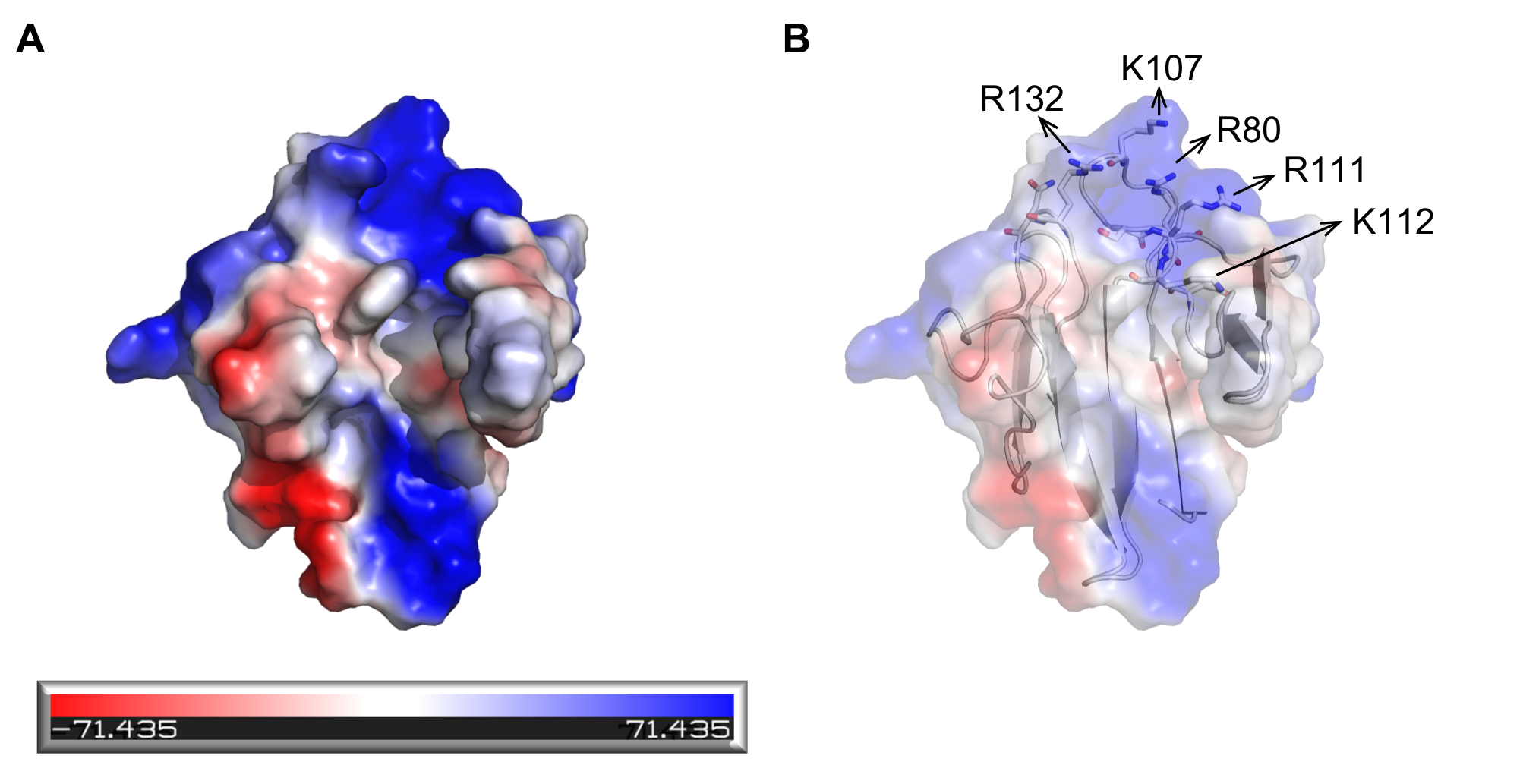

Supplement: S3 Fig — (A) Model of electrostatic potential. Blue indicates positively charged regions. Red indicates negatively charged regions. (B) The positively charged region on the phosphopeptide interaction surface contains residues R80, K107, R111, K112, and R132. See methods section for details on the generation of the Fkh1 structure model. (TIF) [file pgen.1006094.s003.tif]
