# Supplementary material for: Binding of the Fkh1 Forkhead Associated Domain to a Phosphopeptide within the Mph1 DNA Helicase Regulates Mating-Type Switching in Budding Yeast
Source: PLoS Genet. 2016 Jun 3;12(6):e1006094. doi: 10.1371/journal.pgen.1006094 (PMC4892509; doi:10.1371/journal.pgen.1006094)
Supplement: S1 Table — (DOCX) [file pgen.1006094.s004.docx]

**S1 Table. Yeast strains used in this study.** For many assays multiple independent strains were used. For brevity only one is included in this table.

| **Name** | **Description** | **Source** |
| --- | --- | --- |
| PJ69-4A | *MATa trp1-901 leu2-3,112 ura3-52 his3-200 gal4*Δ *gal80*Δ *GAL2-ADE2 LYS2::GAL1-HIS3 met2::GAL7-lacZ* | James 1996 |
| CFY3533 | *MATa ADE2+ RAD5+ CAN1+* | This study |
| CFY3537 | *MATa ADE2+ RAD5+ CAN1+ fkh1*Δ*::HisG* | This study |
| CFY3539 | *MATa ADE2+ RAD5+ CAN1+ mph1*Δ*::KanMX* | This study |
| CFY3549 | *MATa ADE2+ RAD5+ CAN1+ fkh2*Δ*::HisG* | This study |
| CFY3552 | *MATa ADE2+ RAD5+ CAN1+ fkh1*Δ*::HisG fkh2*Δ*::HisG* | This study |
| CFY3886 | *MATa ADE2+ RAD5+ CAN1+ fkh1-R132A* | This study |
| CFY3888 | *MATa ADE2+ RAD5+ CAN1+ fkh1-K107A* | This study |
| CFY3893 | *MATa ADE2+ RAD5+ CAN1+ fkh1-D102A* | This study |
| CFY3894 | *MATa ADE2+ RAD5+ CAN1+ fkh1-R111A* | This study |
| CFY3956 | *MATa ADE2+ RAD5+ CAN1+ fkh1-R80A* | This study |
| CFY3960 | *MATa ADE2+ RAD5+ CAN1+ fkh1-S110A* | This study |
| CFY3963 | *MATa ADE2+ RAD5+ CAN1+ fkh1-S155A* | This study |
| CFY3969 | *MATa ADE2+ RAD5+ CAN1+ fkh1-fha*Δ *(*Δ*50-202)* | This study |
| CFY3971 | *MATa ADE2+ RAD5+ CAN1+ fkh1-N133A* | This study |
| CFY3978 | *MATa ADE2+ RAD5+ CAN1+ fkh1-K112A* | This study |
| CFY3995 | *MATa ADE2+ RAD5+ CAN1+ fkh1-S110A fkh2*Δ*::HisG* | This study |
| CFY4034 | *MATa ADE2+ RAD5+ CAN1+ fkh1-dbd*Δ | Modified from Hollenhorst 2000 |
| CFY4038 | *MATa ADE2+ RAD5+ CAN1+ mph1-2TA* | This study |
| CFY4068 | *MATa ADE2+ RAD5+ CAN1+ Mph1-3FLAG-HIS3 fkh1*Δ*::HisG* | Modified from Chen 2009 |
| CFY4069 | *MATa ADE2+ RAD5+ CAN1+ Mph1-3FLAG-HIS3* | Modified from Chen 2009 |
| CFY4143 | *MATa ADE2+ RAD5+ CAN1+ mph1-2TA-3FLAG-HIS3* | This study |
| CFY4246 | *MATa ADE2+ RAD5+ CAN1+ fkh1-R80A fkh2*Δ*::HisG* | This study |
| CFY4248 | *MATa ADE2+ RAD5+ CAN1+ fkh1-fha*Δ *(*Δ*50-202) fkh2*Δ*::HisG* | This study |
| CFY4249 | *MATa ADE2+ RAD5+ CAN1+ fkh1-D102A fkh2*Δ*::HisG* | This study |
| CFY4251 | *MATa ADE2+ RAD5+ CAN1+ fkh1-K112A fkh2*Δ*::HisG* | This study |
| CFY4252 | *MATa ADE2+ RAD5+ CAN1+ fkh1-S155A fkh2*Δ*::HisG* | This study |
| CFY4254 | *MATa ADE2+ RAD5+ CAN1+ fkh1-K107A fkh2*Δ*::HisG* | This study |
| CFY4257 | *MATa ADE2+ RAD5+ CAN1+ fkh1-R111A fkh2*Δ*::HisG* | This study |
| CFY4258 | *MATa ADE2+ RAD5+ CAN1+ fkh1-R132A fkh2*Δ*::HisG* | This study |
| CFY4262 | *MATa ADE2+ RAD5+ CAN1+ fkh1-N133A fkh2*Δ*::HisG* | This study |
| CFY4267 | *MATa ADE2+ RAD5+ CAN1+ fkh1-fha*Δ *(*Δ*50-202) Mph1-3FLAG-HIS3* | Modified from Chen 2009 |
| CFY4345 | *MATa ADE2+ RAD5+ CAN1+ fkh1-dbd*Δ *fkh2*Δ*::HisG* | Modified from Hollenhorst 2000 |
| CFY4383 | *MATa ADE2+ RAD5+ CAN1+ ade3::GAL-HO HMRα-B re*Δ*::4LexA-Kan arg56::LexAFkh1FHA-LEU2* Δ*ho* | Modified from Li 2012 |
| CFY4387 | *MATa ADE2+ RAD5+ CAN1+ ade3::GAL-HO HMRα-B re*Δ*::4LexA-Kan arg56::LexAFkh1FHA-LEU2* Δ*ho mph1*Δ*::KanMX* | Modified from Li 2012 |
| CFY4394 | *MATa ADE2+ RAD5+ CAN1+ ecm30*Δ*::KanMX* | This study |
| CFY4397 | *MATa ADE2+ RAD5+ CAN1+ ure2*Δ*::KanMX* | This study |
| CFY4400 | *MATa ADE2+ RAD5+ CAN1+ fdo1*Δ*::KanMX* | This study |
| CFY4417 | *MATa ADE2+ RAD5+ CAN1+ ade3::GAL-HO HMRα-B re*Δ*::4LexA-Kan arg56::LexAFkh1FHA-LEU2* Δ*ho mph1-2TA* | Modified from Li 2012 |
| CFY4465 | *MATa ADE2+ RAD5+ CAN1+ gln3*Δ*::KanMX* | This study |
| CFY4500 | *MATa ADE2+ RAD5+ CAN1+ ade3::GAL-HO HMRα-B re*Δ*::4LexA-Kan arg56::LexAFkh1FHA-LEU2* Δ*ho fdo1*Δ*::KanMX* | Modified from Li 2012 |
| CFY4501 | *MATa ADE2+ RAD5+ CAN1+ ade3::GAL-HO HMRα-B re*Δ*::4LexA-Kan arg56::LexAFkh1FHA-LEU2* Δ*ho fdo1*Δ*::KanMX mph1*Δ*::KanMX* | Modified from Li 2012 |
| CFY4509 | *MATa ADE2+ RAD5+ CAN1+ ade3::GAL-HO HMRα-B re*Δ*::4LexA-Kan arg56::LexAFkh1FHA-R80A-LEU2* Δ*ho* | Modified from Li 2012 |
| CFY4511 | *MATa ADE2+ RAD5+ CAN1+ ade3::GAL-HO HMRα-B re*Δ*::4LexA-Kan arg56::LexAFkh1FHA-R80A-LEU2* Δ*ho mph1-2TA* | Modified from Li 2012 |
| CFY4522 | *MATa ADE2+ RAD5+ CAN1+ ade3::GAL-HO HMRα-B re*Δ*::4LexA-Kan arg56::LexAFkh1FHA-LEU2* Δ*ho mph1-Q603D-3FLAG-HIS3* | Modified from Li 2012, Chen 2009 |

**References:**

Chen Y-H, Choi K, Szakal B, Arenz J, Duan X, Ye H, et al. Interplay between the Smc5/6 complex and the Mph1 helicase in recombinational repair. Proc Natl Acad Sci. 2009;106: 21252–21257.

Hollenhorst PC, Bose ME, Mielke MR, Müller U, Fox CA. Forkhead genes in transcriptional silencing, cell morphology and the cell cycle. Overlapping and distinct functions for FKH1 and FKH2 in Saccharomyces cerevisiae. Genetics. 2000;154: 1533–1548.

James P, Halladay J, Craig EA. Genomic libraries and a host strain designed for highly efficient two-hybrid selection in yeast. Genetics. 1996;144: 1425-1436.

Li J, Coïc E, Lee K, Lee C-S, Kim J-A, Wu Q, et al. Regulation of budding yeast mating-type switching donor preference by the FHA domain of Fkh1. PLoS Genet. 2012;8: e1002630.
