# Supplementary material for: Binding of the Fkh1 Forkhead Associated Domain to a Phosphopeptide within the Mph1 DNA Helicase Regulates Mating-Type Switching in Budding Yeast
Source: PLoS Genet. 2016 Jun 3;12(6):e1006094. doi: 10.1371/journal.pgen.1006094 (PMC4892509; doi:10.1371/journal.pgen.1006094)
Supplement: S2 Table — (DOCX) [file pgen.1006094.s005.docx]

**S2 Table. Plasmids used in this study.**

| **Name** | **Description** | **Source** |
| --- | --- | --- |
| pGBDU-C1 | Yeast-2-hybrid vector-Gal4 DNA Binding Domain | James 1996 |
| pGAD-C1 | Yeast-2-hybrid vector-Gal4 Activation Domain | James 1996 |
| pCF577 | pRS426 Fkh1-GBD (GBD in place of FKH1 DBD-used for original 2-hybrid screen) | This study |
| pCF2086 | pGBDU-C1 Mph1 (762-993) (pGBDU-C1 Mph1-Ct) | This study |
| pCF2098 | pGBDU-C1 Fkh1 | This study |
| pCF2099 | pGBDU-C1 Fkh1 (50-291) | This study |
| pCF2101 | pGBDU-C1 Fkh1 (100-291) | This study |
| pCF2106 | pGBDU-C1 Fkh1 (50-202) | This study |
| pCF2113 | pGBDU-C1 Fkh1 (50-158) | This study |
| pCF2183 | pGAD-C1 Fkh1 (50-202) | This study |
| pCF2185 | pGAD-C1 Mph1 (762-993) (pGAD-C1 Mph1-Ct) | This study |
| pCF2203 | pGBDU-C1 Mph1-Ct T785A | This study |
| pCF2205 | pGBDU-C1 Mph1-Ct T776A | This study |
| pCF2297 | pGBDU-C1 Fkh1 R80A | This study |
| pCF2299 | pGBDU-C1 Fkh1 (50-202) R80A | This study |
| pCF2411 | pGBDU-C1 Fkh1 (50-202) A106V | This study |
| pCF2413 | pGBDU-C1 Fkh1 (50-202) R117A | This study |
| pCF2415 | pGBDU-C1 Fkh1 (50-202) S155A | This study |
| pCF2422 | pGBDU-C1 Fkh1 (50-202) K107A | This study |
| pCF2423 | pGBDU-C1 Fkh1 (50-202) R111A | This study |
| pCF2424 | pGBDU-C1 Fkh1 (50-202) R132A | This study |
| pCF2461 | pGBDU-C1 Fkh1 (50-202) N81A | This study |
| pCF2463 | pGBDU-C1 Fkh1 (50-202) D102A | This study |
| pCF2465 | pGBDU-C1 Fkh1 (50-202) N133A | This study |
| pCF2504 | pGBDU-C1 Fkh1 (50-202) T82A | This study |
| pCF2506 | pGBDU-C1 Fkh1 (50-202) D83A | This study |
| pCF2508 | pGBDU-C1 Fkh1 (50-202) N86A | This study |
| pCF2510 | pGBDU-C1 Fkh1 (50-202) K96A | This study |
| pCF2512 | pGBDU-C1 Fkh1 (50-202) K97A | This study |
| pCF2514 | pGBDU-C1 Fkh1 (50-202) N98A | This study |
| pCF2516 | pGBDU-C1 Fkh1 (50-202) S110A | This study |
| pCF2518 | pGBDU-C1 Fkh1 (50-202) K112A | This study |
| pCF2559 | pGAD-C3 Ecm30 (1005-1183) | James 1996 |
| pCF2561 | pGAD-C1 Gln3 (20-189) | James 1996 |
| pCF2568 | pGAD-C2 Ure2 (84-354) | James 1996 |
| pCF2570 | pGAD-C2 Fdo1 (98-342) | James 1996 |
| pCF2571 | pGAD-C1 Smc5 | This study |
| pCF2573 | pGAD-C1 Rfa1 | This study |
| pCF2575 | pGAD-C1 Mhf2 | This study |
| pCF2586 | pGBDU-C1 Mph1-Ct T776AT785A | This study |
| pCF2663 | pGBDU-C1 Mph1 | This study |
| pCF2694 | pGBDU-C1 Mph1 (Δ751-810) | This study |
| pCF2696 | pGBDU-C1 Mph1 (Δ762-993) | This study |
| pCF2697 | pGBDU-C1 Mph1 (751-810) | This study |
| pCF2774 | pGBDU-C1 Fkh1 (Δ50-202) | This study |
| pCF2958 | pET28b-Fkh1 Ct His tag | This study |
| pCF4144 | pGAD-C1 Mph1-Ct N773A | This study |
| pCF4146 | pGAD-C1 Mph1-Ct N773AT785A | This study |
| pCF4148 | pGAD-C1 Mph1-Ct D774A | This study |
| pCF4150 | pGAD-C1 Mph1-Ct D774AT785A | This study |
| pCF4152 | pGAD-C1 Mph1-Ct S775A | This study |
| pCF4153 | pGAD-C1 Mph1-Ct S775AT785A | This study |
| pCF4154 | pGAD-C1 Mph1-Ct E777A | This study |
| pCF4156 | pGAD-C1 Mph1-Ct E777AT785A | This study |
| pCF4157 | pGAD-C1 Mph1-Ct E778A | This study |
| pCF4159 | pGAD-C1 Mph1-Ct E778AT785A | This study |
| pCF4160 | pGAD-C1 Mph1-Ct S782A | This study |
| pCF4161 | pGAD-C1 Mph1-Ct T776AS782A | This study |
| pCF4162 | pGAD-C1 Mph1-Ct L783A | This study |
| pCF4164 | pGAD-C1 Mph1-Ct T776AL783A | This study |
| pCF4166 | pGAD-C1 Mph1-Ct E784A | This study |
| pCF4168 | pGAD-C1 Mph1-Ct T776AE784A | This study |
| pCF4170 | pGAD-C1 Mph1-Ct E786A | This study |
| pCF4172 | pGAD-C1 Mph1-Ct T776AE786A | This study |
| pCF4174 | pGAD-C1 Mph1-Ct D787A | This study |
| pCF4175 | pGAD-C1 Mph1-Ct T776AD787A | This study |
| pCF4177 | pGAD-C1 Mph1-Ct E788A | This study |
| pCF4179 | pGAD-C1 Mph1-Ct T776AE788A | This study |
| pCF4207 | pGAD-C1 Mph1-Ct T776D | This study |
| pCF4209 | pGAD-C1 Mph1-Ct T785D | This study |
| pCF4211 | pGAD-C1 Mph1-Ct T776DT785D | This study |
| pCF4213 | pGAD-C1 Mph1-Ct T776E | This study |
| pCF4215 | pGAD-C1 Mph1-Ct T785E | This study |
| pCF4217 | pGAD-C1 Mph1-Ct T776ET785E | This study |

**References:**

James P, Halladay J, Craig EA. Genomic libraries and a host strain designed for highly efficient two-hybrid selection in yeast. Genetics. 1996;144: 1425-1436.
